# Extensive Ethnolinguistic Diversity in Vietnam Reflects Multiple Sources of Genetic Diversity

**DOI:** 10.1093/molbev/msaa099

**Published:** 2020-04-28

**Authors:** Dang Liu, Nguyen Thuy Duong, Nguyen Dang Ton, Nguyen Van Phong, Brigitte Pakendorf, Nong Van Hai, Mark Stoneking

**Affiliations:** m1 Department of Evolutionary Genetics, Max Planck Institute for Evolutionary Anthropology, Leipzig, Germany; m2 Institute of Genome Research, Vietnam Academy of Science and Technology, Hanoi, Vietnam; m3 Laboratoire Dynamique du Langage, UMR5596, CNRS & Université de Lyon, Lyon, France

**Keywords:** Mainland Southeast Asia, genetic diversity, human admixture, cultural diffusion

## Abstract

Vietnam features extensive ethnolinguistic diversity and occupies a key position in Mainland Southeast Asia. Yet, the genetic diversity of Vietnam remains relatively unexplored, especially with genome-wide data, because previous studies have focused mainly on the majority Kinh group. Here, we analyze newly generated genome-wide single-nucleotide polymorphism data for the Kinh and 21 additional ethnic groups in Vietnam, encompassing all five major language families in Mainland Southeast Asia. In addition to analyzing the allele and haplotype sharing within the Vietnamese groups, we incorporate published data from both nearby modern populations and ancient samples for comparison. In contrast to previous studies that suggested a largely indigenous origin for Vietnamese genetic diversity, we find that Vietnamese ethnolinguistic groups harbor multiple sources of genetic diversity that likely reflect different sources for the ancestry associated with each language family. However, linguistic diversity does not completely match genetic diversity: There have been extensive interactions between the Hmong-Mien and Tai-Kadai groups; different Austro-Asiatic groups show different affinities with other ethnolinguistic groups; and we identified a likely case of cultural diffusion in which some Austro-Asiatic groups shifted to Austronesian languages during the past 2,500 years. Overall, our results highlight the importance of genome-wide data from dense sampling of ethnolinguistic groups in providing new insights into the genetic diversity and history of an ethnolinguistically diverse region, such as Vietnam.

## Introduction

Mainland Southeast Asia (MSEA) is of great interest in terms of ethnolinguistic diversity and deep population history. The early settlement of anatomically modern humans in MSEA dates back to at least 65 thousand years ago (ka) (Bae et al. 2017; Demeter et al. 2017) and is associated with the formation of a hunter-gatherer tradition called Hoabinhian (Higham 2013). Since the Neolithic period, which began about ∼4–5 ka, cultural transitions and diversification have happened multiple times (Edmondson and Gregerson 2007; Blench 2011; Sidwell 2014; Higham 2014; Blench 2017; Bellwood 2018), eventually leading to the extraordinary cultural diversity in present-day MSEA. To date, there are hundreds of ethnolinguistic groups in MSEA, speaking languages belonging to five major language families: Austro-Asiatic (AA), Austronesian (AN), Hmong-Mien (HM), Tai-Kadai (TK), and Sino-Tibetan (ST).

Vietnam occupies a key position in MSEA. It borders China, Laos, and Cambodia and possesses a long coastline, allowing interactions with populations from southern China, MSEA, and Island Southeast Asia (ISEA). Vietnam has a population size of more than 96 million people (www.gso.gov.vn; accessed *the General Statistics Office of Vietnam* in September 2019), comprising 54 official ethnic groups; 110 languages are spoken in the country (Eberhard et al. 2019), and all five language families are represented. Most of these ethnic groups are found in either the southern highlands (mainly the AA and AN groups) or the northern highlands; the latter are especially heterogeneous and include AA, HM, TK, and ST groups (Eberhard et al. 2019). The majority ethnic group in the lowlands is the AA-speaking Kinh, comprising ∼86% of the population (Dang et al. 2010; Eberhard et al. 2019), hence the genetic studies of Vietnamese to date have focused mainly on the Kinh (Vu-Trieu et al. 1997; Ivanova et al. 1999; Pischedda et al. 2017; Le et al. 2019). The genetic profiles of the other 53 official ethnic groups remain largely unexplored, leaving a substantial gap in our understanding of their genetic relationships and history.

The presence of five language families in Vietnam suggests diverse origins for this ethnolinguistic diversity. Although linguistic and archeological evidence suggest several population movements into Vietnam (Edmondson and Gregerson 2007; Blench 2011; Matsumura and Oxenham 2014; Sidwell 2014; Higham 2014; Blench 2017; Bellwood 2018).

Genetic studies can inform on this question. For example, ancient genome studies have provided indications of demic diffusion, in that the present-day AA groups in MSEA show evidence of admixture involving Hoabinhian hunter-gatherers and the ancestors of Neolithic East Asians (Lipson et al. 2018; McColl et al. 2018). Another study of the mitochondrial DNA (mtDNA) control-region of the AN-speaking Cham suggested that they are likely to have resulted from language and culture shift of the indigenous AA-speaking Mon-Khmer populations to an AN language and culture (Peng et al. 2010). A later study generated mtDNA control-region data from the Kinh and four ethnic minority groups and identified different haplogroup profiles among the AA, TK, HM, and ST groups (Pischedda et al. 2017). More recent studies analyzed complete mtDNA genome sequences (Duong et al. 2018; Macholdt et al. 2020) and partial sequences of the male-specific portion of the Y chromosome (MSY) (Macholdt et al. 2020) from the Kinh and 16 ethnic minority groups and further confirmed the diverse genetic profile in Vietnam. However, genome-wide studies, which can provide more resolution and additional insights into population relationships and history, are so far limited to the Kinh (Pischedda et al. 2017; Le et al. 2019).

To further investigate the genetic diversity in Vietnam, we generated genome-wide single-nucleotide polymorphism (SNP) data from 22 Vietnamese ethnolinguistic groups, speaking languages that encompass all five families in MSEA. We incorporate published data and analyze the allele and haplotype sharing within the Vietnamese groups and between them and both nearby modern populations and nearby SEA ancient samples. Our results provide new insights into the genetic diversity of these ethnolinguistically diverse groups, including their recent interactions and demography.

## Results

### Overview of Population Structure

**Fig. 1.**
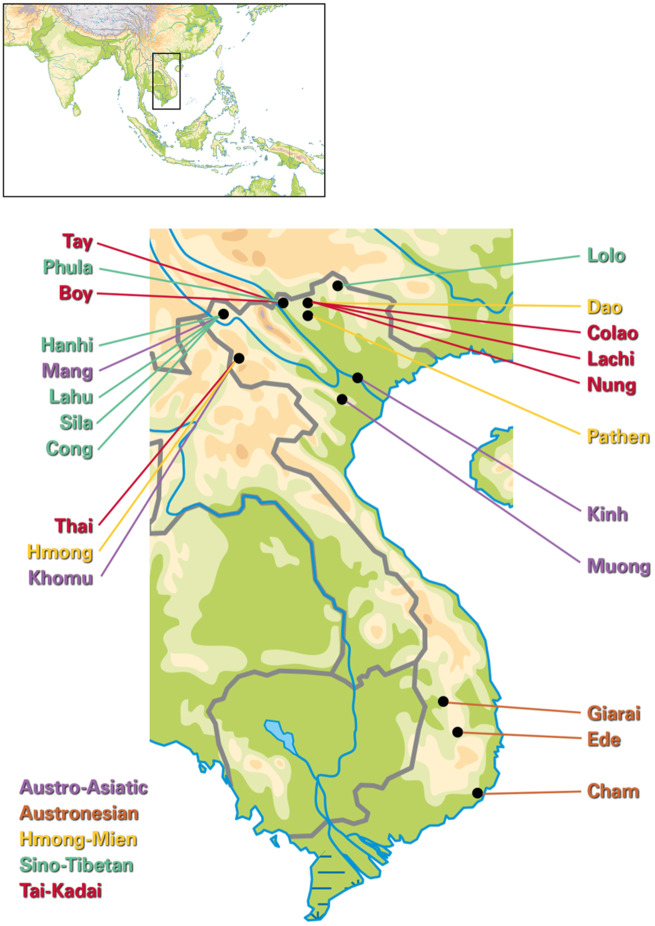
Map of the sampled Vietnamese ethnolinguistic groups. Dots denote the median of the sampling geographic coordinates per group.

**Fig. 2.**
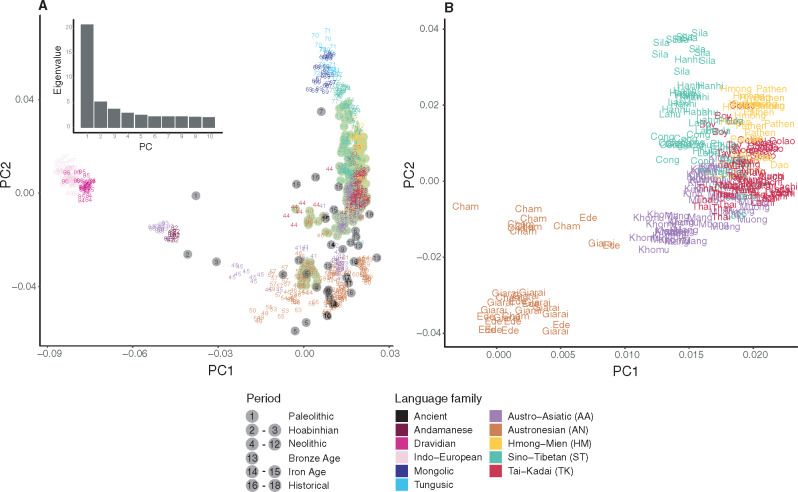
PCA analyses. (*A*) PCA analysis of 712 individuals and 33,666 SNPs, with individuals colored according to language families. The more isolated modern populations (Mamanwa, Mlabri, Onge, and Jehai) and the ancient samples were projected. The eigenvalues from PC1 to PC10 are shown in the top left corner. Ancient samples are shown as gray dots, whereas the present-day Vietnamese are shown as light green dots. Each population and ancient sample is numbered according to supplementary table S1, Supplementary Material online. (*B*) Vietnamese populations only, zoomed-in from (*A*).

We genotyped individuals from 22 Vietnamese ethnolinguistic groups (fig. 1) and merged the data with data from nearby modern populations and ancient samples (supplementary fig. S1, Supplementary Material online). We started by applying principal components analysis (PCA) and the clustering algorithm ADMIXTURE (Alexander et al. 2009) to explore population structure. With other East Asian (EA) and Indian groups included (fig. 2*A* and supplementary fig. S2, Supplementary Material online, with the populations numbered according to supplementary table S1, Supplementary Material online), the strongest signal (i.e., variation along PC1) separates most Indian groups from the EA groups, with the Indian groups Kharia (#83 in the figures) and Onge (#82) placed between them. The ancient EA sample from Tianyuan (#1) and the Hoabinhian samples from Pha Faen (#2) and Gua Cha (#3) are projected between the Onge and the Jehai (#45) from Malaysia. The addition of PC2 further spreads out the EA groups, with the Mongola and northern Chinese groups (#67–73) at one end and ISEA groups (#45–58) at the other. With respect to language family, the ST, HM, and TK groups are mostly separated from AA and AN groups. Neolithic SEA ancient samples (#4–12) are mostly projected near the AA and AN groups, except that the sample from Oakaie (#7) is projected near the ST groups and other northern Chinese groups. The Bronze (#13), Iron age (#14–15), and historical (#16–18) samples are shifted more toward the present-day Vietnamese (#19–40).

Within modern Vietnamese groups, individuals from the same language family are mostly placed together (fig. 2*B*). There is some overlapping of individuals from different language families, except that AN groups are distinct from the others, closer to the AA-speaking Cambodian (#41), Htin Mal (#42), Mlabri (#43), and many ISEA populations, all of whom speak AN languages (fig. 2*A* and supplementary table S1, Supplementary Material online). When considering additional PCs, the Ede and Giarai are strongly differentiated on PC3 from all other groups, except for the AA-speaking Khomu and Mang (supplementary fig. S3, Supplementary Material online). Additional PCs tend to highlight the distinctiveness of the Mang, ST-speaking Sila and TK-speaking Colao and Lachi.

**Fig. 3.**
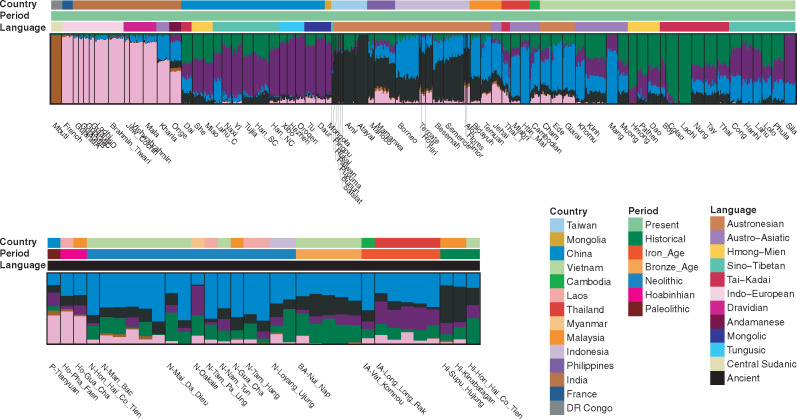
ADMIXTURE analyses. ADMIXTURE result for *K* = 6, which minimized the cross-validation error (supplementary fig. S4, Supplementary Material online). Regions are labeled at the top, whereas population/ancient sample names are labeled at the bottom. The color bar denotes the language families and time periods for the modern and ancient samples, respectively.

We next performed an ADMIXTURE analysis and found that the lowest cross-validation error occurs at *K* = 6 (supplementary fig. S4, Supplementary Material online). Under the model of *K* = 6 (fig. 3, with estimates of each source in each Vietnamese group and ancient sample in supplementary table S2, Supplementary Material online), there is a brown source present only in the Mbuti; a pink source enriched in both the French and Indian groups; a blue source enriched in AA-speaking groups and in AN-speaking groups from Indonesia, Malaysia, and Vietnam; a black source enriched in AN-speaking groups from Taiwan, Philippines, and Indonesia; a purple source appearing in all of the Chinese groups and enriched in the Vietnamese ST groups; and a dark green source absent before *K* = 6 appearing in the southern Chinese groups and enriched in Vietnamese HM and TK groups. In general, Vietnamese groups show diverse genetic profiles with variable amounts of the dark green, purple, blue, and black sources. The Vietnamese AN groups are notable in that the amount of AN-related black source in them does not surpass other Vietnamese groups and in having higher frequencies of the pink source (12% on average in AN groups compared with a maximum of 1.5% in the other Vietnamese groups). With respect to the ancient samples, the pink source is enriched in the Tianyuan and Hoabinhian samples, whereas the blue source is enriched in the Neolithic samples. The blue source decreases in ancient samples younger than the Neolithic, with a concomitant increase of the green, purple, or black sources. Specifically, the green and black sources increase in the Bronze Age and historical samples in Vietnam. The black source also increases in the historical samples from Malaysia, whereas the purple source is enriched not only in the Iron Age sample from Long Long Rak (Thailand) but also in the Neolithic sample from Oakaie (Myanmar).

Overall, there is considerable variation among the Vietnamese groups in the frequencies of some of the specific sources. In particular, Vietnamese AN groups are more similar in this analysis to some groups from Malaysia, Thailand, and Cambodia, and to the Neolithic ancient samples (except for the sample from Oakaie), than to other Vietnamese groups. Also, the HM-speaking Hmong and the TK-speaking Colao and Lachi stand out in lacking the black and blue sources, whereas the ST-speaking Sila and the AA-speaking Mang lack the black and green sources. The remaining Vietnamese groups present fairly similar profiles (albeit with some variation in the frequencies of specific sources) that are also similar to the Dai from southern China.

Although higher values of *K* are associated with higher cross-validation errors, they can nevertheless provide additional insights (supplementary figs. S5 and S6, Supplementary Material online). At *K* = 7, the French get their own source, which is practically absent in all of the Vietnamese individuals, and confirms that the pink source in the Vietnamese AN-speaking groups is likely shared deep ancestry with Indian groups. At *K* = 8, many ISEA populations have high frequencies of the peach source, which is at highest frequency in Alor and Timor. This source decreases the black source present in the SEA groups and the Vietnamese AN groups. At *K* = 9 and 10, the Mang and Lachi get their own source, respectively. At *K* = 11, the Sila obtain their own source, which also shows up in the ST groups in both China and Vietnam. At *K* = 12 and 13, the Atayal and Colao get their own source, respectively. At *K* = 14, the Htin Mal get their own source, which also shows up in many Neolithic samples. Finally, at *K* = 15, the Hmong get their own source, which is also present in all of the HM groups.

### Investigation of Population Relationships and Demography

The above analyses (PC and ADMIXTURE) are descriptive analyses that provide an overview of the relationships of the populations analyzed. To further explore and quantify these relationships, we used outgroup *f*3 and *f*4 statistics to identify ancestry sharing based on allele sharing, and identity by descent (IBD) approaches to investigate demography and recent contact based on haplotype sharing.

#### *Outgroup* f*3*

Higher values of the outgroup *f*3 statistic indicate more shared drift, and hence a closer relationship, between two test populations since their divergence from the outgroup population.

**Fig. 4.**
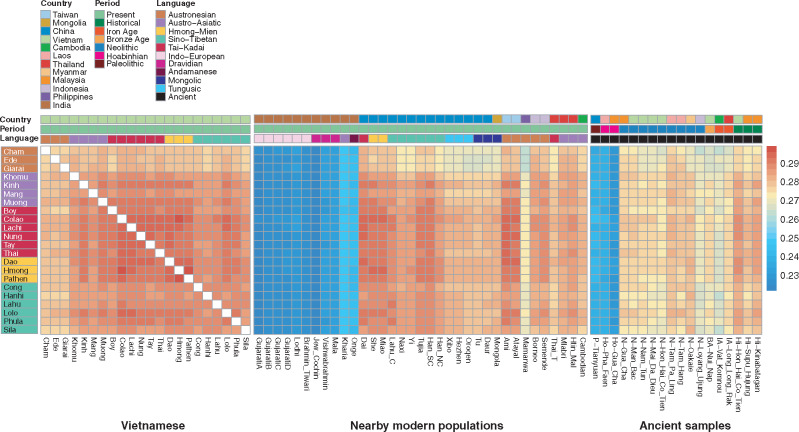
Heatmap of outgroup *f*3 profiles. A heatmap based on the values of the *f*3 statistic for all pairs of populations/ancient samples, using Mbuti as the outgroup. Shown are comparisons among the Vietnamese groups (left), with the nearby modern populations (middle), and with the ancient samples (right). The three different color bars at the top denote separately the countries, time periods, and language families, according to the key. The Vietnamese group labels are also shaded according to language family.

We first compared the *f*3 results within Vietnamese groups (fig. 4 and supplementary fig. S7, Supplementary Material online). The AN groups are again most distant from others and also show more shared drift with some non-AN groups than with each other. The AA groups exhibit two distinct sharing profiles: The Mang/Khomu have relatively low levels of shared drift with all other Vietnamese groups, whereas the Muong/Kinh have higher levels of sharing with each other and with some TK, HM, and ST groups. The TK and HM groups share the most with each other, with Muong/Kinh, and with the ST-speaking Lolo and Phula.

Next, we investigated the relationships between Vietnamese and neighboring modern populations (fig. 4 and supplementary fig. S8, Supplementary Material online). Vietnam ethnolinguistic groups overall tend to show the closest relationships with Taiwanese and southern Chinese groups. Consistent with the PCA results (supplementary fig. S2, Supplementary Material online), the Vietnamese groups are mostly distant from Indian populations. For the AN groups, the Ede and Giarai exhibit higher *f*3 values with the AA-speaking Mlabri and Htin Mal, whereas Cham shows more sharing with the AN-speaking Ami and TK-speaking Dai. The AA groups can again be separated into the Mang/Khomu versus, Muong/Kinh, with the former showing relatively more sharing with the AA-speaking Htin Mal and Mlabri but less with the AN-speaking Ami and TK-speaking Dai than the latter. Overall, the HM and TK groups generally seem to share more with the TK-speaking Dai, HM-speaking Miao and She, ST-speaking Tujia and Han, and the AN-speaking Ami and Atayal, than with the Vietnamese AA, AN, and ST groups. The ST groups exhibit high *f*3 values with several southern Chinese populations, particularly the ST-speaking Chinese Lahu. Similar outgroup *f*3 profiles are obtained when the French are used as an outgroup instead of the Mbuti (supplementary fig. S9, Supplementary Material online).

When compared with ancient samples (fig. 4 and supplementary fig. S10, Supplementary Material online), all the Vietnamese groups exhibit high *f*3 values with the historical samples from Hon Hai Co Tien (Vietnam) and Kinabatagan (Malaysia), except for the AN groups. The *f*3 values normalized to range from 0 to 1 tend to be especially high (>0.95) with the historical sample from Hon Hai Co Tien. The AN-speaking Ede/Giarai as well as the AA-speaking Mang/Khomu show higher *f*3 values with the Neolithic samples from Tam Pa Ling (Laos), Tam Hang (Laos), Gua Cha (Malaysia), and Man Bac (Vietnam). The *f*3 values with the Neolithic sample from Oakaie (Myanmar), Bronze Age sample from Nui Nap (Vietnam), and Iron Age sample from Long Long Rak (Thailand) are generally high with all groups except the AN groups. The smallest *f*3 values are those with the Paleolithic sample from Tianyuan and the Hoabinhian samples, and the *f*3 values with these samples show little variation among Vietnamese groups.

#### Identity by Descent

**Fig. 5.**
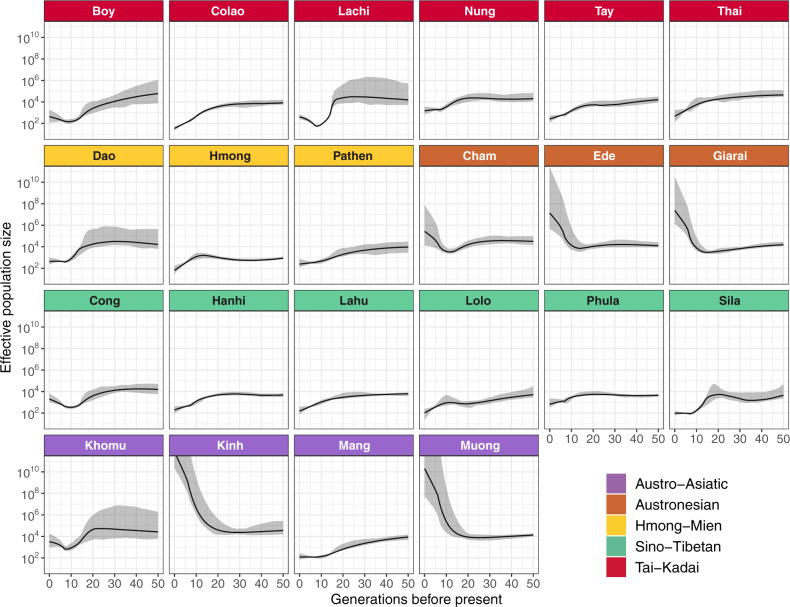
Effective population size of Vietnamese ethnolinguistic groups over the past 50 generations. Each panel depicts an ethnolinguistic group. The panels are colored according to language family; 95% confidence intervals are shaded in gray.

We next investigated interactions within/between populations within the past ∼3 ka by analyzing IBD (Ralph and Coop 2013; Al-Asadi et al. 2019). The number and length of IBD segments shared within a population provides further insights into population demography (Browning SR and Browning BL 2015; Browning et al. 2018; Ceballos et al. 2018; Severson et al. 2019). The Hmong, Pathen, Lachi, Boy, Colao, Mang, Lolo, and Sila all show elevated levels of within population IBD sharing, whereas the Kinh have the lowest level (supplementary fig. S11, Supplementary Material online). We used the IBD sharing within each population to directly estimate recent changes in effective population size (fig. 5), that is, within the past 50 generations (Browning SR and Browning BL 2015). The Boy, Lachi, Dao, Sila, Cong, and Khomu are inferred to have experienced bottleneck events, whereas the AA-speaking Kinh and Muong have undergone population expansions beginning around 15–20 generations (∼450–600 years) ago. The three AN groups have also undergone a slight reduction in population size ∼450–600 years ago, followed by population expansions ∼300–450 years ago. Other populations show no obvious bottleneck events but an overall decrease in size; in particular, the Colao, Hmong, Lolo, and Mang have very small effective population sizes.

**Fig. 6.**
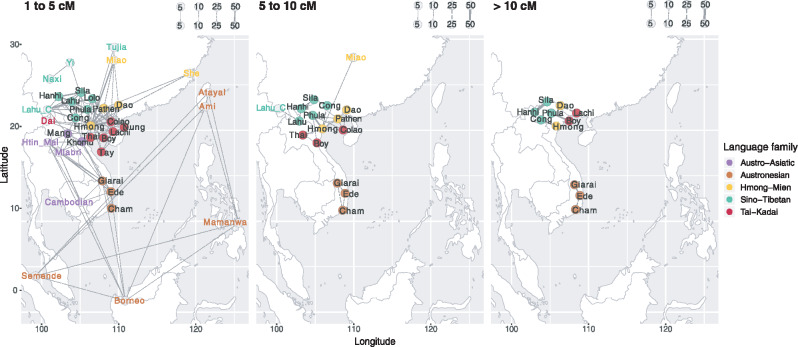
IBD sharing between populations. Network visualizations of the mean of summed IBD lengths shared between populations, with identified IBD blocks in the range of 1–5 cM (oldest), 5–10 cM, and over 10 cM (youngest). We focus on the sharing involving Vietnamese groups. The signals were enriched by requiring an average of at least two shared IBD blocks per pair of individuals (4 for the range of 1–5 cM). Each node stands for a population, and each edge indicates the IBD sharing between populations. The nodes of the Vietnamese groups are jittered for visibility and the labels of the neighboring populations are colored according to language family. The width of each edge is proportional to the mean of the summed IBD length, with the scale (cM) provided in the top-right portion of each figure (dashed line type for ≤25 cM).

Although IBD sharing within populations provides insights into population size changes, IBD sharing between populations provides insights into recent contact and/or shared ancestry; the longer the shared IBD blocks, the more recent the interaction. We analyzed IBD blocks in three categories: 1–5, 5–10, and >10 cM (fig. 6); these correspond very roughly to time intervals of 1,500–2,500 years ago, 500–1,500 years ago, and 0–500 years ago, respectively (Ralph and Coop 2013). The oldest (smallest) shared IBD segments show wide interaction and/or recent common ancestor sharing of Vietnam ethnolinguistic groups with neighboring populations and within their language families; these become more and more localized in the younger (larger) shared IBD segments. In the range of 5–10 cM, the only sharing between Vietnamese and others is Vietnamese Lahu with Chinese Lahu, and Hmong with Miao; among Vietnamese groups, the HM, TK, and ST groups are intermixed, whereas the AN groups share exclusively with each other. In the range of over 10 cM, sharing is limited to only a few localized pairs between ST, HM, and TK groups as well as within the AN groups. Notably, the AA-speaking Kinh and Muong do not share any IBD blocks with any other group, irrespective of the size of the blocks.

#### f*4 Statistics*

**Fig. 7.**
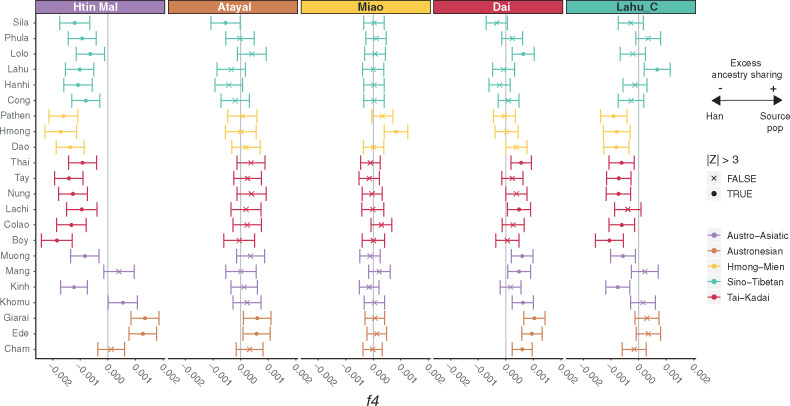
f4 statistics comparing Vietnamese groups to representative source populations. *Z*-scores are for f4(W, southern Han Chinese; Y, Mbuti), where W is the source population (panel labels) and Y is the Vietnamese group (label on the *y* axis). Bars give three standard errors in each direction. Significant negative values indicate that Han Chinese share more ancestry with Y, whereas significant positive values indicate that W shares more ancestry with Y. The vertical gray lines denote 0. The panels are colored according to language family.

We further investigated the relationships of Vietnamese groups with representative source populations for each language family. Based on the *f*3 and IBD-sharing results, we selected the Htin Mal (AA), Atayal (AN), Miao (HM), Dai (TK), and Chinese Lahu (ST) as the representative source populations for the five language families in Vietnam. We then calculated *f*4 statistics of the form *f*4(Source populations, southern Han Chinese; Vietnamese, Mbuti) to test if each Vietnamese group shares any excess ancestry with any of the representative source populations, compared with the southern Han Chinese. Significantly positive *Z*-scores indicate excess shared ancestry between the Vietnamese group and the source population, whereas significantly negative *Z*-scores indicate excess shared ancestry between the Vietnamese group and southern Han Chinese. The resulting Vietnamese *f*4 profiles are heterogeneous within each language family (fig. 7). The AA-speaking Khomu and AN-speaking Ede and Giarai show significant excess ancestry sharing with the AA-speaking Htin Mal. All other Vietnamese groups show excess ancestry sharing with southern Han Chinese, except for the AA-speaking Mang and the AN-speaking Cham, which show no excess shared ancestry. With the AN-speaking Atayal as the source, the only significant sharing is between Atayal and the AN-speaking Ede and Giarai and between southern Han Chinese and the ST-speaking Sila. With the HM-speaking Miao as the source, the only significant sharing is between the HM-speaking Hmong and the Miao. With the TK-speaking Dai as the source, there is significant sharing between the Dai and the ST-speaking Lolo, the TK-speaking Thai and Lachi, the AA groups except the Kinh, and all of the AN groups. Finally, with the ST-speaking Chinese Lahu as the source, there is significant sharing between them and the ST-speaking Vietnamese Lahu. In contrast, the southern Han Chinese share ancestry with all of the HM groups, all of the TK groups (except Lachi), and with the AA-speaking Muong and Kinh. Overall, these results are consistent with the other analyses that suggest different sources for the genetic diversity in different Vietnamese ethnolinguistic groups.

When we used ancient samples as the source population in this *f*4 statistic, no Vietnamese group shares excess ancestry with any ancient sample (supplementary fig. S12, Supplementary Material online). Instead, practically all of the Vietnamese groups share excess ancestry with southern Han Chinese; the few exceptions, in which there is no excess sharing between the Vietnamese group and either the southern Han Chinese or the source population, involve various of the AN groups, Khomu, Mang, and/or Sila with the Neolithic samples and the Iron Age sample from Vat Komnou. Also, many Vietnamese groups share no excess ancestry with southern Han Chinese in the comparisons with historical samples.

The population structure analyses suggested a shift in the affinities of the ancient samples, with pre-Neolithic/Neolithic samples more similar to AA and AN groups, and more recent samples exhibiting more similarities to TK, HM, and ST groups (figs. 2 and 3). To further investigate this, we used Mlabri, Htin Mal, Borneo, Ami, and Mamanwa as a combined representative source of the AA and AN groups, and Dai, Miao, Chinese Lahu, southern Han Chinese, and northern Han Chinese as a combined representative source of the TK, HM, and ST groups, and then computed *f*4 statistics of the form *f*4(TK/HM/ST, AA/AN groups; Ancient samples, Mbuti). We found that the AA and AN groups indeed shared excess ancestry with the Hoabinhian sample from Pha Faen, most of the Neolithic samples except for the samples from Oakaie (which shares excess ancestry with the TK, HM, and ST groups) and Nam Tun, and the historical samples from Supu Hujung and Kinabatagan (supplementary fig. S13, Supplementary Material online). This result supports the shift in affinities of ancient samples that was observed in the population structure analyses. To avoid any potential attraction to deep outgroups and/or noise from DNA damage patterns in ancient samples, we used the French as a closer outgroup and restricted the analyses with the ancient samples to transversions. This reduced the number of SNPs from 361,327 to 64,126, and correspondingly many of the *Z*-scores became nonsignificant; however, the overall trends are similar (supplementary figs. S14 and S15, Supplementary Material online).

### Admixture Graph Inference

Based on the sharing profiles revealed by the *f*3, IBD, and *f*4 analyses, we next built admixture graphs for Vietnamese groups from each language family. Admixture graphs, which depict a history of population divergence and admixture events, use either a combination of *F*-statistics or a covariance matrix of the allele frequencies (Nielsen 2018). We first applied TreeMix (Pickrell and Pritchard 2012) and AdmixtureBayes (Nielsen 2018) to systematically survey (i.e., without supervision) the potential admixture graphs based on the covariance matrix of allele frequencies, and we further tested if these graphs are accepted in qpGraph (Patterson et al. 2012), using a combination of *F*-statistics. Before building the graph for each language family, we first built a tree with all the Vietnamese groups, the representative source populations used in the *f*4 analyses, the Onge, selected ancient samples, and the Mbuti as an outgroup (supplementary fig. S16, Supplementary Material online). We found that all of the ancient samples fall outside the Vietnamese clade, except that the historical sample from Kinabatagan shares an ancestor with the clade of the ST groups and an admixture source from the lineage leading to the AN-speaking Atayal. The AN groups are placed outside the clade of other Vietnamese groups; the former is close to the Neolithic samples from Tam Pa Ling and the AA-speaking Htin Mal. The AA-speaking Kinh and Muong and the ST-speaking Phula and Lolo are close to the HM and TK groups rather than to other groups from the same language family. The HM-speaking Dao is closer to the TK groups compared with other HM groups, whereas the TK-speaking Colao is placed in the clade of HM groups.

**Fig. 8.**
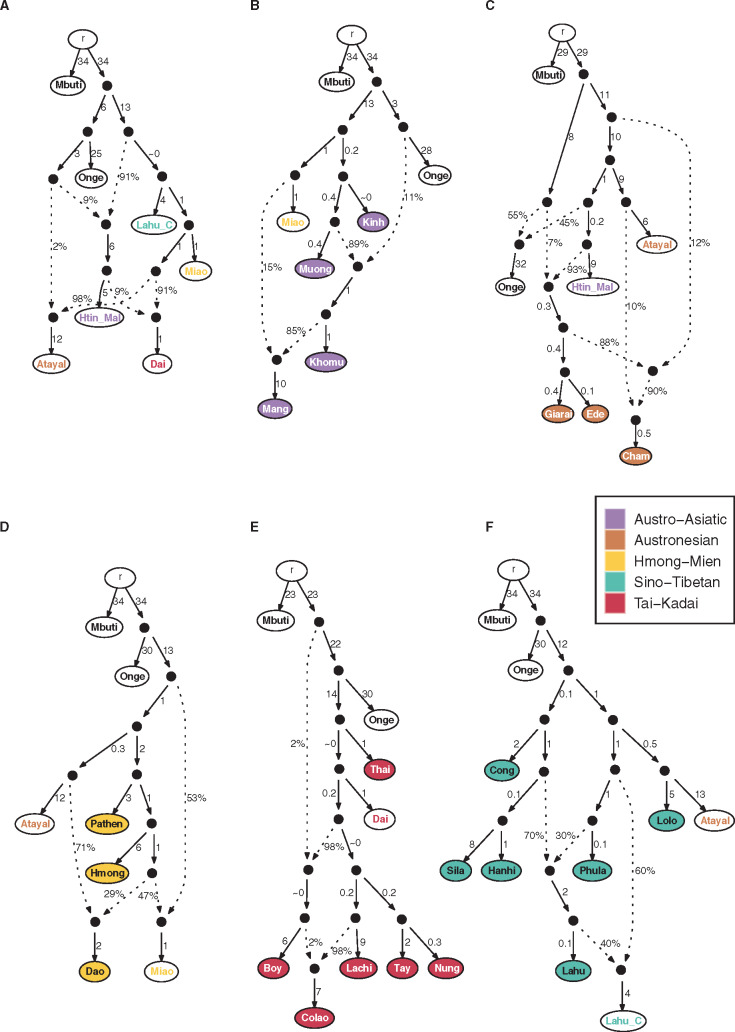
Admixture graphs of the Vietnamese groups, for each language family. The best-fitting admixture graphs are shown for the backbone populations and for the Vietnamese groups, done separately for each language family. The node r denotes the root. White nodes denote backbone populations. Backbone population labels and Vietnamese nodes are colored according to language family. Dashed arrows represent admixture edges, whereas solid arrows are drift edges reported in units of FST × 1,000. (*A*) backbone populations (worst-fitting *Z* = −2.189). (*B*) AA groups (worst-fitting *Z* = −2.263). (*C*) AN groups (worst-fitting *Z* = −1.258). (*D*) HM groups (worst-fitting *Z* = −1.462). (*E*) TK groups (worst-fitting *Z* = 2.381). (*F*) ST groups (worst-fitting *Z* = −2.656).

On a local scale, we started with a backbone graph with the representative source populations used in the *f*4 analyses, the Onge, and the Mbuti as an outgroup, for further investigating the admixture graphs by each language family. The best-fitting backbone graph (worst-fitting *Z* = −2.189) shows that the first split separates the Onge from a branch leading to the ST-speaking Chinese Lahu and the HM-speaking Miao (fig. 8*A*; TreeMix results in supplementary fig. S17, Supplementary Material online). All other groups are derived via admixture events. The AA-speaking Htin Mal have ∼9% ancestry from the ancestor of the Onge and 91% ancestry from an ancestor of the Chinese Lahu and Miao. The AN-speaking Atayal have ∼2% ancestry from this same Onge ancestor, and 98% ancestry from a source related to the Miao. Finally, the TK-speaking Dai have ∼91% ancestry from this same Miao-related source, and ∼9% ancestry from an ancestor of the Htin Mal (and thereby also share some ancestry with Onge and Atayal). This graph includes an edge that has ∼0 length, which introduces some uncertainty about the topology; to try to resolve this further, we investigated alternative graphs and found one without any edges of length ∼0 that is slightly worse but still acceptable (worst-fitting *Z* = −2.235). This graph maintains the same branching order for the Chinese Lahu, Miao, and Dai (supplementary fig. S18*A*, Supplementary Material online).

The best-fitting admixture graph (worst-fitting *Z* = −2.263) for the Vietnamese AA groups (fig. 8*B*; Treemix results in supplementary fig. S19, Supplementary Material online) supports the division noted in previous analyses for the Kinh/Muong versus the Khomu/Mang. The former share an ancestor with the Miao, whereas the latter are admixed from sources related to the Onge and the Muong (similar to the Htin Mal in the backbone graph), with the Mang in addition having ∼15% Miao-related ancestry. This graph does not include the AA-speaking Htin Mal as their inclusion leads to an unacceptable graph (worst-fitting *Z* = −3.642), but even so this graph retains the Kinh/Muong versus Khomu/Mang division (supplementary fig. S18*B*, Supplementary Material online).

The best-fitting graph for the AN groups (worst-fitting *Z* = −1.258) shows different histories for the Giarai and Ede versus the Cham (fig. 8*C*; TreeMix results in supplementary fig. S20, Supplementary Material online). The Giarai/Ede have ∼7% ancestry from an ancestor of the Onge, and ∼93% ancestry from an ancestor of the Htin Mal, whereas the Cham have ancestry from an ancestor of the Atayal and Htin Mal, an ancestor specifically of the Atayal, and an ancestor of the Giarai/Ede (thereby contributing Onge-related and additional Htin Mal-related ancestry). This graph is quite complex with four admixture events, so we investigated if the number of admixture events could be reduced. We found that the admixture event leading to the Onge could be eliminated, as the resulting graph has almost the same *Z*-score (worst-fitting *Z* = −1.265; supplementary fig. S18*C*, Supplementary Material online). This graph still retains three admixture events for the AN groups; we could not find an acceptable graph that eliminated any of these admixture events (all graphs investigated with two admixture events have worst-fitting *Z*-scores with an absolute value >6).

For the HM groups, the best-fitting graph (worst-fitting *Z* = −1.462) indicates that the Hmong and Pathen share an ancestor with the Atayal, whereas the Dao are admixed from an ancestor of the Atayal and a node derived from the ancestor of the Hmong (fig. 8*D*; TreeMix results in supplementary fig. S21, Supplementary Material online). In this graph, the Miao are modeled as having admixed ancestry from the same node that contributes to the Dao that is related to the ancestor of the Hmong, and an ancestor of the Atayal/Pathen/Hmong. This graph does not include the Dai as a potential source of TK ancestry; adding them results in an acceptable graph (worst-fitting *Z* = 2.627) in which the Dai share an ancestor with the Atayal; the Miao are not modeled as admixed but share ancestry with the Hmong, Atayal, and Dai; the Pathen are admixed between an ancestor of the Hmong and an ancestor of the Atayal/Dai; and the Dao are admixed between the same or a closely related ancestor of the Hmong (our data are insufficient to distinguish between these two possibilities) and an ancestor of the Dai (supplementary fig. S18*D*, Supplementary Material online). Thus, this graph suggests that the Dao have TK-related ancestry rather than AN-related ancestry.

In the best-fitting graph (worst-fitting *Z* = 2.381), the TK groups Thai, Lachi, Nung, and Tay form a clade with the Dai (fig. 8*E*; TreeMix results in supplementary fig. S22, Supplementary Material online). The Boy have admixed ancestry with an ancestor of this clade and the Onge, and an ancestor of the Lachi/Nung/Tay, whereas the Colao have admixed ancestry involving an ancestor of the Boy and an ancestor of the Lachi. Considering the close relationship between the TK and HM groups shown in the other analyses, we tried to include the Miao as a potential source of HM ancestry. Inclusion of the Miao (supplementary fig. S18*E*, Supplementary Material online) results in a worst-fitting *Z* of 3.049 and has essentially the same relationships except that the Colao are modeled as mixed between an ancestor of the Lachi and an ancestor of the Miao; the Miao share an ancestor with the Boy.

All of the ST groups (except the Lahu) form a clade together with the Atayal in the best-fitting graph (worst-fitting *Z* = −2.656), with the Lolo most closely related to the Atayal (fig. 8*F*; TreeMix results in supplementary fig. S23, Supplementary Material online). The Vietnamese Lahu have admixed ancestry from an ancestor of the Hanhi/Sila and an ancestor of the Phula, and the Chinese Lahu are modeled as having admixed ancestry from an ancestor of the Vietnamese Lahu and an ancestor of the Phula. Considering the excess ancestry sharing between the Lolo and the TK-speaking Dai shown in the *f*4 analyses, we tried to include the Dai as a potential source of TK ancestry. Inclusion of the Dai results in a worst-fitting *Z* of 3.499 (supplementary fig. S18*F*, Supplementary Material online). In this graph, the Dai share an ancestor with the Atayal and show less drift to the Lolo than the Atayal do (6 vs. 17.5). There are minor rearrangements in the relationships of some of the ST groups, but the Vietnamese and Chinese Lahu are both still modeled as admixed.

## Discussion

### Extensive Genetic Diversity among Vietnamese Groups

In this study, we have generated and analyzed genome-wide SNP data from 22 ethnolinguistic groups in Vietnam encompassing all five language families in MSEA (supplementary table S1, Supplementary Material online). We found extensive genetic diversity among Vietnamese groups in the PCA and ADMIXTURE analyses (figs. 2 and 3 and supplementary figs. S3 and S5, Supplementary Material online). Hence, the majority group Kinh, which have been the focus of previous studies, may not reflect the total Vietnamese diversity, although we note that our sample of Kinh is relatively small and may not reflect the true genetic diversity of the Kinh. Overall, the AN groups are distinct from the others but closest to the AA groups (fig. 2). The HM, TK, and ST groups share more ancestry with present-day southern Chinese groups, and the former two are more closely related to each other (figs. 2–4 and 6). By incorporating ancient samples from SEA and China, we have shown that the AA ancestry rose in the Neolithic period, followed by an increase of AN, HM/TK, or ST ancestry (according to the region) in later periods (fig. 3 and supplementary table S2, Supplementary Material online). This population turnover from the Neolithic to later periods, with additional Chinese-related ancestry, is consistent with the archeological and linguistic studies (Edmondson and Gregerson 2007; Sidwell 2014; Higham 2014; Blench 2017) but contradicts a previous study, based on much more limited sampling, that claimed a largely indigenous origin for Vietnamese groups (Le et al. 2019). As discussed in more detail below, the overall Vietnamese genetic diversity likely reflects multiple waves of ancestry from the Neolithic to later periods. These correlate somewhat (but not completely) with the language families, as we now discuss for each language family.

### Austro-Asiatic

The possible origins of the AA family include southern China, MSEA, or India (Sidwell 2014). It is thought to be the oldest language family in MSEA, which emerged after the Hoabinhian tradition ∼4–5 ka (Sidwell 2014). Ancient genome studies have suggested that the present-day AA groups in MSEA are descendants of Hoabinhian hunter-gatherers and ancestral EAs from southern China admixing during the Neolithic farming expansion (Lipson et al. 2018; McColl et al. 2018). Consistent with this scenario, we find that the indigenous AA groups Htin Mal and Khomu have 9% and 11% ancestry from the Hoabinhian hunter-gatherers and 91% and 89% ancestry from the ancestors of southern Chinese, respectively (fig. 8*A* and *B*). The AA-speaking Mang are closer to the Khomu compared with the Kinh and Muong, but they also share ancestry with the ST-speaking Chinese Lahu in the TreeMix analysis (supplementary fig. S19, Supplementary Material online), and they share ancestry with the HM-speaking Miao in the qpGraph analysis (fig. 8*B*). This ancestry sharing with ST-speaking Chinese Lahu could reflect the proximity of the Mang to ST groups (fig. 1). In contrast, the AA-speaking Kinh and Muong share more drift with HM and TK groups than with other AA-speaking groups (fig. 4). In particular, they are not estimated as having ancestry from the Hoabinhians in the admixture graph, in contrast to the Mang and Khomu (fig. 8*B* and supplementary fig. S19, Supplementary Material online). This is consistent with previous suggestions that the Kinh and Muong may be related to the Dong Son culture and have ancestors from southern China (Dang et al. 2010; Blench 2017) but contradicts one recent study stating that the Kinh appear to be an indigenous SEA group with less EA ancestry (Le et al. 2019). However, the latter study included only the Kinh and Thai as SEA groups and the Han, Korean, and Japanese as EA groups. It is likely that our inclusion of many more SEA and Chinese groups, and more detailed sampling of Vietnamese ethnolinguistic groups, provides a more accurate picture of their relationships.

As the Kinh and Muong have the highest census size of Vietnamese groups (Dang et al. 2010; Eberhard et al. 2019), it seems likely that they have interacted extensively with each other as well as with HM and TK groups. However, although we found that the Khomu and the Mang share IBD blocks with each other and with ST and AN groups, we did not find any strong IBD sharing between the Kinh and Muong and other groups (fig. 6). This is consistent with the uniparental marker data, which show no haplotype sharing between the Kinh and other groups (Macholdt et al. 2020). Moreover, we observed exponential population expansions in the Kinh and Muong, compared with population contractions in the Khomu and Mang, ∼20 generations (∼600 years) ago (fig. 5). We caution that our estimation of effective population size is likely to be uncertain for populations with large effective population sizes in recent generations, due to the assumption of a constant growth rate, and insufficient sample sizes for accurate estimation (Browning SR and Browning BL 2015; Browning et al. 2018). This lack of sufficient sampling may also dilute the signals of between population IBD sharing, and hence the Kinh and Muong may have had some recent contact with HM and TK groups, even if this is not visible in the IBD-sharing analysis.

### Austronesian

The origin of the AN family is proposed to be Taiwan (Gray et al. 2009; Ko et al. 2014; Sidwell 2014). The expansion of the AN groups into ISEA is dated ∼3–4 ka (Gray et al. 2009; Sidwell 2014), whereas the emergence of the AN family in MSEA is thought to have happened ∼2.5 ka (Peng et al. 2010; Sidwell 2014; Higham 2014). Previous linguistic studies thus suggested that the introduction of the AN family into MSEA was via migration from ISEA after the initial expansion from Taiwan (Edmondson and Gregerson 2007; Blench 2011; Sidwell 2014). In particular, the ancestors of the Cham are thought to have come from ISEA, probably Indonesia, and they established the Kingdom of Champa and dominated southern Vietnam during the 2nd to mid-15th century (Edmondson and Gregerson 2007; Blench 2011; Sidwell 2014; Blench 2017). In contrast, genetic studies of mtDNA suggested that the emergence of the Cham was primarily mediated by cultural diffusion (Peng et al. 2010). The other two AN groups, Ede and Giarai, have high frequencies of mtDNA haplogroups which are specific to Vietnam but absent in Taiwanese AN speakers (Duong et al. 2018), and also have a high frequency of mtDNA but no partial MSY haplotype sharing with each other (Macholdt et al. 2020). We find that the AN groups actually share less ancestry with Taiwan AN groups than do most other groups from Vietnam; however, Cham do share slightly more ancestry with the Taiwanese AN groups than do the Ede and Giarai, whereas the Ede and Giarai share slightly more ancestry with the AN-speaking Borneo and AA-speaking Htin Mal and Mlabri (figs. 4 and 6). Moreover, the admixture graph results show that the Ede and Giarai can be modeled as having exclusively AA-associated ancestry, whereas the Cham have ∼10% ancestry from an ancestor of the AN-speaking Atayal (fig. 8*C* and supplementary fig. S20, Supplementary Material online). To sum up, the pattern we have observed in AN groups likely reflects the ancestors of the Cham coming from ISEA and interacting extensively with AA groups, which resulted in the Cham acquiring substantial AA-related ancestry. These interactions led other AA groups to shift to AN languages (e.g., the Ede and Giarai). Thus, the AN-speaking groups of Vietnam do not reflect a purely cultural process for the spread of AN languages, but rather both migration and cultural diffusion. However, we should emphasize that additional sampling of Central and Southern Vietnamese ethnolinguistic groups is needed to fully document their interactions with the groups we have studied.

In the IBD results, we observe that ∼1.5–2.5 ka the Vietnamese AN groups are mostly connected with neighboring AA groups and with an AN-speaking group from Borneo (fig. 6), which has been shown to have excess AA-related ancestry (Lipson et al. 2014). We also observe strong IBD sharing between the Ede and Giarai over the entire size range of IBD blocks, which is consistent with the uniparental data for these two groups (Macholdt et al. 2020). Additionally, the AN-speaking groups underwent population expansion around 300–450 years ago (fig. 5). A similar population expansion was inferred for the Giarai and Ede based on partial Y chromosome sequences (Macholdt et al. 2020; the Cham were not included in this study). However, the inferred timing of population expansion based on the Y chromosome is much older (∼2,500 and ∼7,500 years ago for the Ede and Giarai, respectively) and was suggested to be possibly linked to the spread of the Dong Son culture (Macholdt et al. 2020). Furthermore, mtDNA genome sequences from the Giarai and Ede did not show any signal of expansion (Macholdt et al. 2020). Given the uncertainty with dating events based on molecular genetic data, it may be that the same expansions are reflected in the autosomal and uniparental marker data. Alternatively, the uniparental markers may lack sufficient resolution to detect more recent expansions. Since the time of expansion of AN groups based on genome-wide data is close to that of the Kinh and Muong, we suggest that these events may be linked.

### Hmong-Mien and Tai-Kadai

Both the HM and TK families are thought to have originated in what is now southern China (and possibly also northern Vietnam for the TK family), and the beginning of their separate migrations into MSEA dates to ∼2.5 ka (Edmondson and Gregerson 2007; Sidwell 2014). The TK and AN proto-languages might be related (Blench 2011; Blench 2017), and TK groups from Thailand have been shown to be related to ANs based on modeling of mtDNA genome sequences (Kutanan et al. 2018). We have also found that the AN-speaking Atayal is placed in the clade of TK groups (supplementary figs. S16, S17, and S22, Supplementary Material online). The early TK, HM, ST, and AA groups are thought to have interacted in what is now southern China (Blench 2011; Blench 2017). It has also been suggested that ancient tribes in southern China, the Baiyue, might be composed of several proto-AA, HM, and TK groups living together (Lee 2012). Compared with the AA and ST, closer interactions between the HM and TK have been shown in genetic studies using uniparental (Macholdt et al. 2020) and insertion/deletion data (He et al. 2019). A recent study further pointed out that Hmongic and Mienic groups from southern China demonstrate different genomic affinities to ST and TK groups, respectively (Xia et al. 2019). We have also found that the Vietnamese HM and TK groups are closely related. Among them, the HM-speaking Dao in particular share more drift and, based on IBD sharing, have more recent interactions with nearby TK groups, especially Colao and Lachi (figs. 4 and 6 and supplementary fig. S18*D*, Supplementary Material online). The Pathen also live close to the TK groups but share more drift and IBD blocks with the Hmong (figs. 4 and 6). This could be explained by the fact that the Hmong and Pathen speak languages that belong to the Hmongic branch of the family and thus might have a more recent common ancestor, whereas the Dao language belongs to the Mienic branch (Eberhard et al. 2019). In contrast, the TK-speaking Colao share more with the HM groups, especially with the Hmong (figs. 4 and 6). The Colao and Hmong show strong IBD sharing, but this does not extend to the range of segments >10 cM. This indicates that their interactions might have ceased in the past 500 years or so (Ralph and Coop 2013; Al-Asadi et al. 2019), which could be due to population decline in both of them around this time (fig. 5). The languages spoken by the Colao and Lachi both belong to the Kra branch of the TK family (Eberhard et al. 2019), hence we suspect that the initial interaction was between early Kra and Mienic groups. Overall, the interactions we identify between the HM and TK groups are consistent with linguistic studies (Blench 2011; Lee 2012; Blench 2017) and genetic studies using uniparental (Macholdt et al. 2020) and insertion/deletions data (He et al. 2019).

### Sino-Tibetan

The ST family originated in northern China ∼7 ka (Sagart et al. 2019; Zhang et al. 2019) and then started to move southward into MSEA ∼3 ka (Sidwell 2014). To further investigate their genetic relationships, we used the Chinese Lahu as the source of ST-related ancestry in Vietnam, even though they show substantial frequencies of the AA-related source in the ADMIXTURE analysis (fig. 3). However, the Chinese Lahu do not display any strong signals of attraction to the AA groups in other analyses (figs. 4, 6, 7, and 8*A* and supplementary fig. S16, Supplementary Material online), and they show stronger affinity to the Vietnamese ST groups in outgroup *f*3 and IBD analyses than other neighboring ST groups (fig. 6 and supplementary fig. S9, Supplementary Material online). Compared with HM and TK groups, the ST groups form a relatively independent and isolated cluster (figs. 2, 6, and 8*F*). Yet, the Lolo and Phula share more drift with the HM and TK groups than do the other ST groups (figs. 4 and 7). In particular, the Lolo are modeled as sharing ancestors with the TK-speaking Dai and AN-speaking Atayal in the admixture graph analysis (fig. 8*F* and supplementary fig. S18*F*, Supplementary Material online). The Lolo and Phula live at lower elevations than the other ST groups, and the Phula live close to several HM and TK groups (fig. 1). Although most of the ST groups show strong IBD sharing with each other, the Phula also share IBD blocks with the HM-speaking Hmong and the TK-speaking Boy in the recent time period (fig. 6). Although the ST-speaking Cong do not show strong shared drift with the HM and TK groups, they do share IBD blocks with the HM-speaking Hmong over the entire size range (fig. 6). This not only agrees with the genomic affinity between Hmongic and ST groups suggested recently (Xia Z-y, Yan S, Wang C-C, Zheng H-X, Zhang F, Liu Y-C, Yu G, Yu B-X, Shu L-L, Jin L, unpublished data. Inland-coastal bifurcation of southern East Asians revealed by Hmong-Mien genomic history. Available from: https://www.biorxiv.org/content/10.1101/730903v1, last accessed November 22, 2019.) but also indicates more recent interactions between the ST and HM groups, within the past few hundred years.

## Conclusion

We have analyzed newly generated genome-wide SNP data for the majority group Kinh plus 21 smaller ethnic groups from Vietnam. These ethnolinguistic groups speak languages that encompass the five major language families in MSEA. Our study shows extensive genetic diversity of the Vietnamese ethnolinguistic groups that is associated with heterogeneous ancestry sharing profiles in each language family. In contrast to previous studies suggesting a largely indigenous origin of the Vietnamese, we find evidence for extensive contact, over different time periods, between Vietnamese and other groups. However, the linguistic diversity is not completely in agreement with genetic diversity. In particular, the HM and TK groups in Vietnam demonstrate extensive interactions with populations speaking languages belonging to different families. Moreover, different AA groups show different affinities with other ethnolinguistic groups (e.g., the AA-speaking Mang show affinities with ST-speaking Chinese Lahu), whereas the AN groups likely reflect language shift involving AA groups. This study highlights the importance of dense sampling of ethnolinguistic groups, combined with genome-wide data from both extant and ancient sources, to gain insights into the history of an ethnolinguistically diverse region such as Vietnam.

## Materials and Methods

### Sample Information

We sampled 259 male Vietnamese individuals (supplementary table S1, Supplementary Material online) belonging to 22 ethnic groups that speak languages belonging to the five language families in Vietnam. Specifically, the ethnic groups consist of four AA-speaking groups (Khomu, Kinh, Mang, and Muong), three AN-speaking groups (Cham, Ede, and Giarai), three HM-speaking groups (Dao, Hmong, and Pathen), six ST-speaking groups (Cong, Hanhi, Lahu, Lolo, Phula, and Sila), and six TK-speaking groups (Boy, Colao, Lachi, Nung, Tay, and Thai). The mtDNA genome (Duong et al. 2018; Macholdt et al. 2020) and partial MSY sequences (Macholdt et al. 2020) for most of these individuals, from 17 of the 22 ethnic groups, were published previously. The median of the geographic coordinates of the sampling locations per population are shown in figure 1. The name, language affiliation, and census size of the ethnic groups included in this project were based on *the General Statistics Office of Vietnam* (www.gso.gov.vn; accessed April 2019 and the 2009 Vietnam Population and Housing census) and the Ethnologue (Eberhard et al. 2019). All sample donors gave written informed consent, and this research received ethical clearance from the Institutional Review Board of the Institute of Genome Research, Vietnam Academy of Science and Technology (No. 4-2015/NCHG-HDDD) and from the Ethics Commission of the University of Leipzig Medical Faculty.

### Genotyping Data Set Information

All sampled individuals were genotyped on the Affymetrix Axiom Genome-Wide Human Origins array (Patterson et al. 2012). We kept only autosomal markers for our analyses, which contain 587,360 markers on the hg19 version of the human reference genome coordinates. In order to study ethnolinguistic history in Vietnam on a spatial-temporal scale, we merged both modern (Reich et al. 2011; Patterson et al. 2012; Lazaridis et al. 2014; Qin and Stoneking 2015) and ancient (Yang et al. 2017; Lipson et al. 2018; McColl et al. 2018) published data from populations within and around MSEA (supplementary fig. S1 and table S1, Supplementary Material online). The ancient DNA data were retrieved from the following studies with all information included and their alleles were obtained through pseudo-haploid strategies (Yang et al. 2017; Lipson et al. 2018; McColl et al. 2018). Ancient samples were labeled by their excavation site and time period, with P: Paleolithic, Ho: Hoabinhian, N: Neolithic, BA: Bronze Age, IA: Iron Age, and Hi: Historical. Data merging was done by mergeit from EIGENSOFT version 7.2.1 (Patterson et al. 2006). Positions with more than two variants or that were inconsistent between two data sets were excluded. For data genotyped on the Affymetrix 6.0 array, we first converted the genomic coordinates from hg18 to hg19 using CrossMap version 0.3.1 (Zhao et al. 2014) and extracted the intersection of markers with our Vietnamese data set using the intersect command in bedtools version 2.25.0 (Quinlan and Hall 2010) before merging. However, incorporating data genotyped on the Affymetrix 6.0 array greatly decreased the number of informative sites due to the low number of intersecting markers (∼60,000), and we therefore only included the Affymetrix 6.0 data in population structure analyses. Similarly, incorporating ancient DNA data also greatly decreased the number of informative sites due to missing data, so we excluded the ancient samples from the phasing and IBD analyses. For quality control, we first checked individual relatedness using KING version 2.1.6 (Manichaikul et al. 2010) and removed one from each pair of individuals with first degree of kinship. After that, we examined the global and within population missing site numbers using the missing command in PLINK version 1.90b5.2 (Purcell et al. 2007). We removed modern individuals with more than 5% global missing data, and ancient individuals with <15,000 informative sites. Then, we excluded variant sites in modern samples with more than 5% global missing data, or 50% missing data within a population. We also used PLINK to perform Hardy–Weinberg equilibrium tests within populations and excluded variant sites with *P* value < 0.00005. The number of individuals and sites for the filtered data used for different analyses is provided in supplementary table S3, Supplementary Material online.

### Population Structure Analyses

We used PCA and ADMIXTURE version 1.3.0 (Alexander et al. 2009) to visualize how the populations cluster. For both methods, variants were pruned beforehand for linkage disequilibrium using PLINK, excluding one variant from pairs with *r*^2^ > 0.4 within windows of 200 variants and a step size of 25 variants. We performed PCA by computing eigenvalues only from the less isolated modern populations and then projecting the more isolated modern populations (Mamanwa, Mlabri, Onge, and Jehai) and the ancient samples, using smartpca from EIGENSOFT with “lsqproject” and “autoshrink” options. We performed heatmap visualization of downstream PCs using the pheatmap package in R version 3.6.0. For running the ADMIXTURE program, we also first estimated the allele frequency of the inferred ancestral populations (i.e., P parameter) using the less isolated modern populations and then projected the more isolated modern populations and the ancient samples with the -P option. From *K* = 2 to *K* = 15, we performed 100 replicates for each *K* with random seeds. Finally, we used pong version 1.4.7 (Behr et al. 2016) to visualize the top 20 highest likelihood ADMIXTURE replicates for the major mode at each *K*. The mean and standard error of the ancestry proportions at *K* = 6 (with the lowest cross-validation error), shown in supplementary table S2, Supplementary Material online, were calculated based on the values of all the individuals within a population from the highest likelihood replicate for Vietnamese groups and for ancient samples with multiple individuals from the same excavation site and time period. For ancient samples with only one individual from an excavation site and time period, we ran 1,000 bootstrap replicates to calculate the standard error using ADMIXTURE with -B parameter and a random seed corresponding to the replicate with highest likelihood.

### Allele Sharing Analyses

We used admixr version 0.7.1 (Petr et al. 2019) to compute *f*3- and *f*4-statistics from ADMIXTOOLS version 5.1 (Patterson et al. 2012), with significance assessed through block jackknife resampling across the genome. Outgroup *f*3-statistics of the form *f*3(X, Y; Outgroup) were used to measure the shared drift between populations X and Y since their divergence from the outgroup. We performed heatmap visualization of *f*3 profiles using the pheatmap package in R. *f*4-statistics of the form *f*4(W, X; Y, Outgroup) were used to formally test whether W or X shares more ancestry with population Y. We used Mbuti as the outgroup for all analyses; to ensure there is no excess shared ancestry between any test population and the outgroup, we also repeated the outgroup *f*3-statistics with French as the outgroup. To avoid attraction to deep outgroups and minimize potential noise from DNA damage patterns in ancient samples, we performed an additional set of *f*4-statistics using French as the outgroup and only transversions.

### Data Phasing

We used SHAPEIT version 2.r904 (Delaneau et al. 2012, 2013, 2014) with a reference panel and recombination map from the 1000 Genome Phase3 (1000 Genomes Project Consortium et al. 2015) to phase the modern samples. For the reference panel we used the East Asia and South Asia populations with KHV (Kinh in Ho Chi Minh City, Vietnam) excluded. To check the consistency of sites and strands between the reference panel and our data set, we ran SHAPEIT with -check option before phasing and excluded markers failing this check. For phasing, the accuracy of SHAPEIT can be increased by increasing the number of iterations and conditioning states on which haplotype estimation is based (Browning SR and Browning BL 2011). We used options –burn 10, –prune 10 and –main 30 for iteration number with 500 conditioning states, leaving other parameters as default.

### IBD Analyses

We used refinedIBD (Browning BL and Browning SR 2013) to identify shared IBD blocks between each pair of individuals and homozygous-by-descent blocks within each individual. We considered both identified IBD and homozygous-by-descent blocks as IBD blocks in our analyses, which have been called pairwise shared coalescence segments in a previous study (Al-Asadi et al. 2019). Then, we merged IBD blocks within a 0.6-cM gap and allowed only one inconsistent genotype between the gap and block regions using the program merge-ibd-segments from BEAGLE utilities (Browning SR and Browning BL 2007; Browning et al. 2018). These results were used to create four data sets based on the length of identified IBD blocks: 1–5 cM, 5–10 cM, over 10 cM, and at least 2 cM. The first three were used to compare the IBD sharing between populations in different time periods (Ralph and Coop 2013; Al-Asadi et al. 2019), whereas the last one was used to investigate IBD sharing within each population (Browning SR and Browning BL 2015; Browning et al. 2018). To summarize the IBD sharing, we summed up the total number and length of IBD blocks for each individual pair and calculated the population median and mean for each data set. We used the network approach in Cytoscape version 3.7.1 (Shannon et al. 2003) to visualize the results and kept the pairs with at least two shared blocks (4 for the range of 1–5 cM) to reduce noise and false positives. To estimate effective population size, we ran IBDNe (Browning SR and Browning BL 2015; Browning et al. 2018) using shared blocks of at least 2 cM within each population, and only extracted the estimated population size numbers within 50 generations ago, as previously suggested for SNP array data (Browning SR and Browning BL 2015). A generation time of 30 years (Fenner 2005) was used to convert generations to years.

### Admixture Graph Analyses

We used admixture graphs to model population histories that fit the genomic data. We separated our Vietnamese data by language family and modeled the admixture graph, together with related source populations, for each family. We first modeled a global admixture graph with the related present-day source populations, ancient samples, and all the Vietnamese groups. These present-day source populations were chosen based on excess ancestry sharing in the *f*4 analyses. Only the ancient samples with <65% missing data were used here in order to have at least 20,000 SNPs for the model estimation. As the ancient samples are not closely related to the Vietnamese groups in the global admixture analysis, and their inclusion decreases the number of SNPs while increasing the complexity of the modeling, we decided to use only the present-day source populations for dissecting the Vietnamese admixture graph. We first modeled an admixture graph with only the related modern source populations, which we call the backbone populations. For each language family and the backbone populations, we pruned the SNPs as we did in the population structure analyses and calculated allele frequencies with PLINK. Using the covariance of the allele frequency profiles as input, we first ran TreeMix version 1.12 (Pickrell and Pritchard 2012) with 0–3 migration events and ten independent runs, and selected the topology with the highest likelihood for further investigation. We also checked and confirmed that the likelihood and topologies of these ten runs are mostly similar, which indicates that the model estimation has reached convergence. Next, we used AdmixtureBayes (Nielsen 2018) to estimate the top ten posterior admixture graphs, based on the covariance of the allele frequency profiles. When more populations are added to the model, more steps will be needed for the Markov chain Monte Carlo to converge. We hence limited the maximum number of populations to 11, for which the model can converge and finish in a reasonable time. To do so, we selected suitable combinations of source populations for each language family, based on the topology showing the lowest standard error in the TreeMix residual plots with three migration events. We used 300,000 Markov chain Monte Carlo steps for each AdmixtureBayes run with stop criteria stopping the run if the summaries of effective sample size are all above 200. We then used the estimated graphs as input for qpGraph from ADMIXTOOLS to test the goodness of fit of the graphs. We accepted the graph as a good fit when the absolute value of the *Z*-score of the worst *f*4 statistic output by qpGraph was <3. For the cases where we failed to find a fit, we adjusted the source populations based on the *f*4 outliers output by qpGraph. Then, we used the –subnodes option in AdmixtureBayes to calculate the posterior of the adjusted subsets and tested the results again in qpGraph. We iterated these procedures until we were able to fit graphs for all of the five language families as well as only the source populations. We ran qpGraph with parameters outpop: NULL, blgsize: 0.05, forcezmode: YES, diag: 0.0001, bigiter: 6, hires: YES, and lambdascale: 1.

## Data Availability

To comply with the informed consent under which the samples were obtained, we make the data available upon request by asking the person requesting the data to agree in writing to the following restrictions: 1) The data will only be used for studies of population history, 2) the data will not be used for medical or disease-related studies, or for studies of natural selection, 3) the data will not be distributed to anyone else, 4) the data will not be used for any commercial purposes, and 5) no attempt will be made to identify any of the sample donors.

## Supplementary Material

Supplementary data are available at *Molecular Biology and Evolution* online.

## Supplementary Material

msaa099_Supplementary_Data
