# Supplementary material for: Extensive Ethnolinguistic Diversity in Vietnam Reflects Multiple Sources of Genetic Diversity
Source: Mol Biol Evol. 2020 Apr 28;37(9):2503–19. doi: 10.1093/molbev/msaa099 (PMC7475039; doi:10.1093/molbev/msaa099)
Supplement: msaa099_Supplementary_Data [file msaa099_supplementary_data.zip › msaa099-Suppl_Data/03.FigS1-23_resubmit2.pdf]

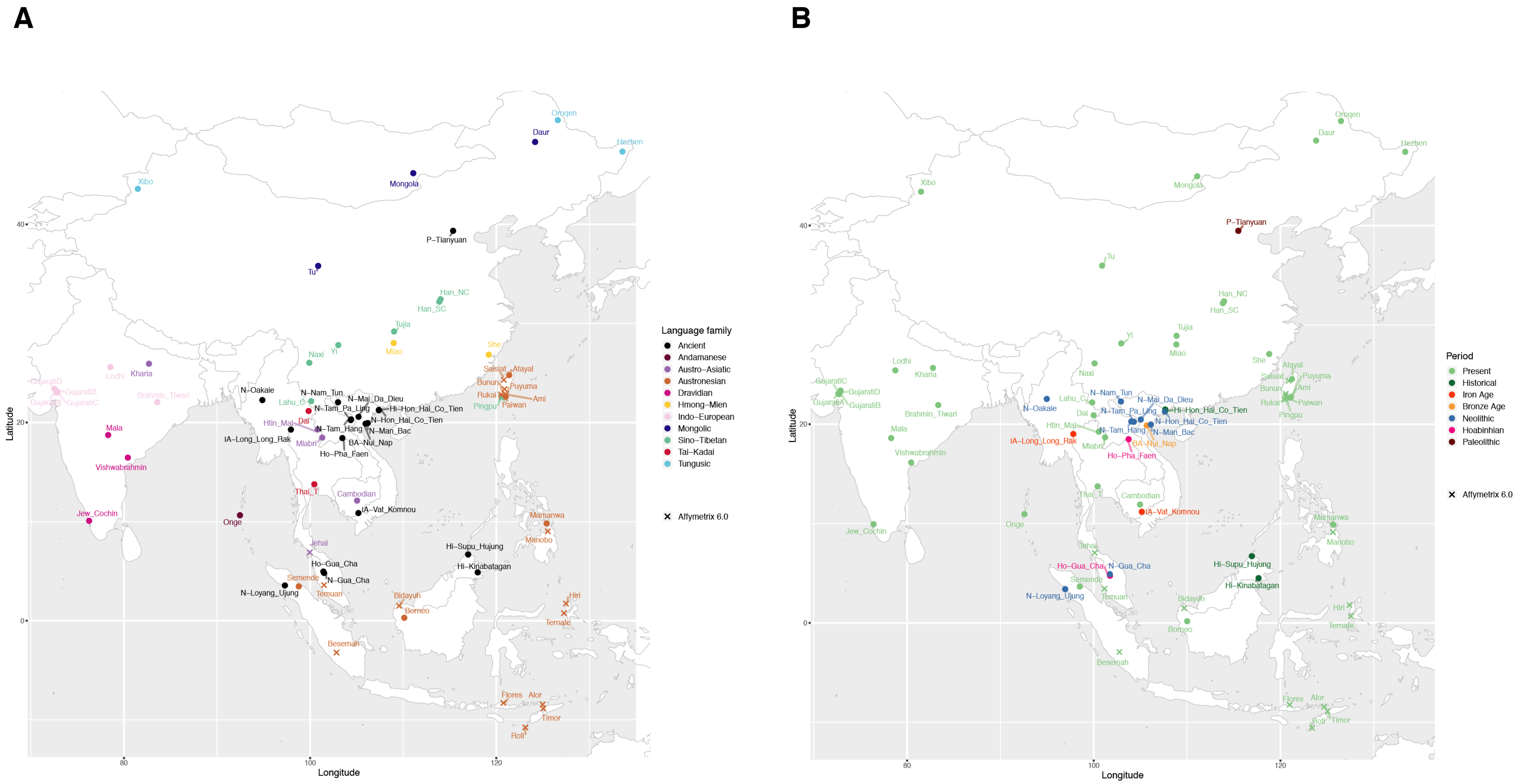

Fig. S1. Sample information map.

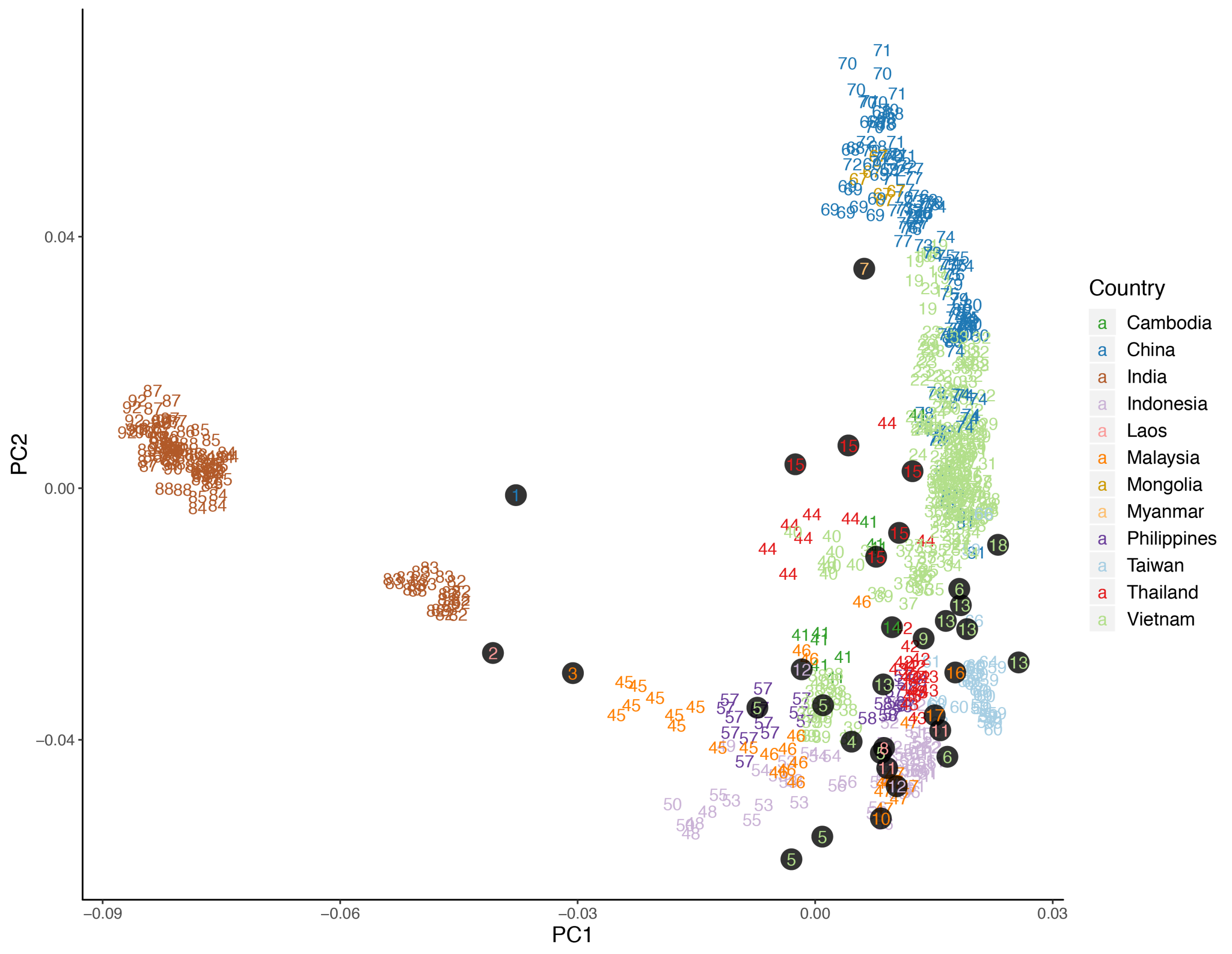

Fig. S2. PCA colored by countries.

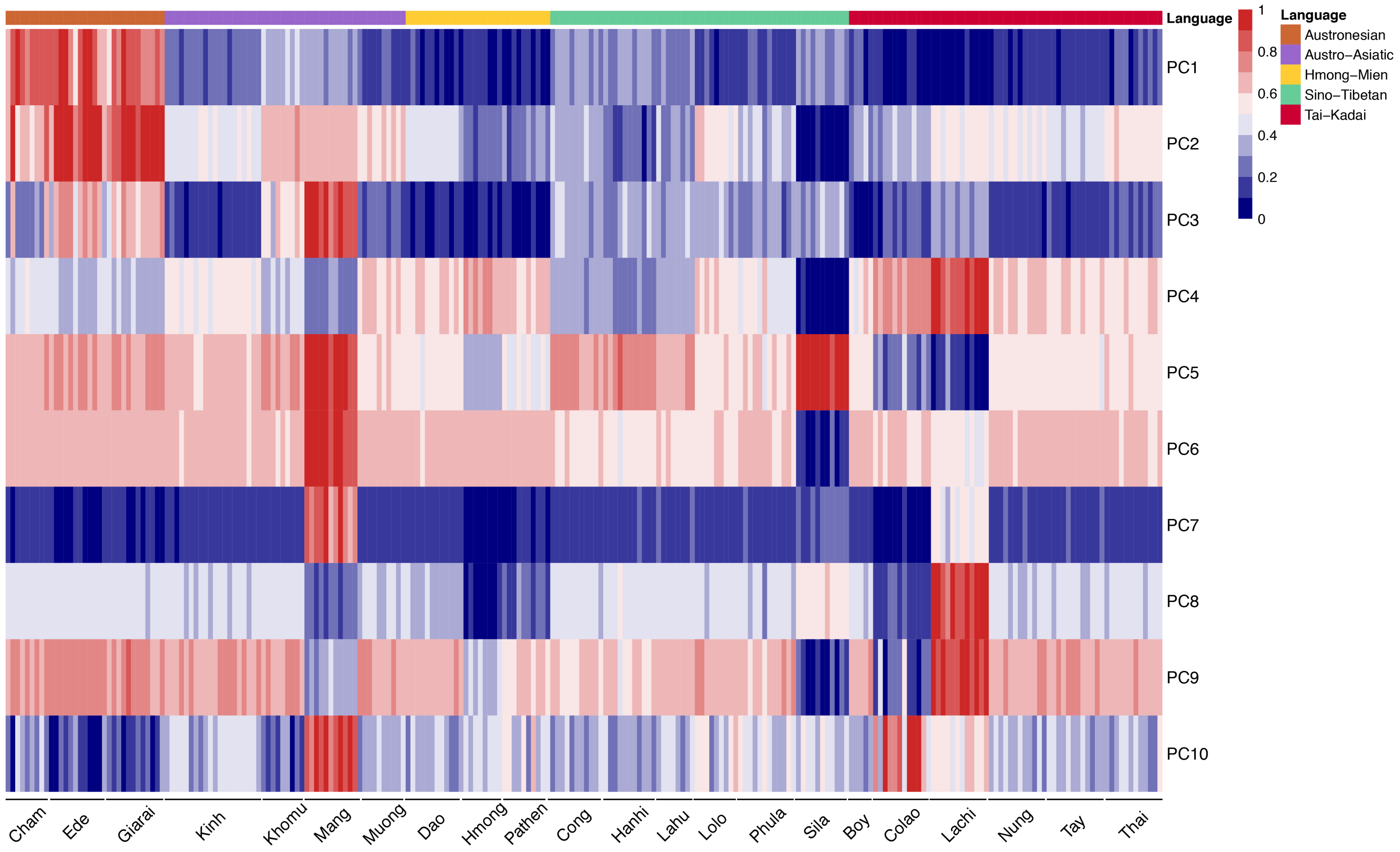

Fig. S3. Heatmap visualization of PC1 to PC10.

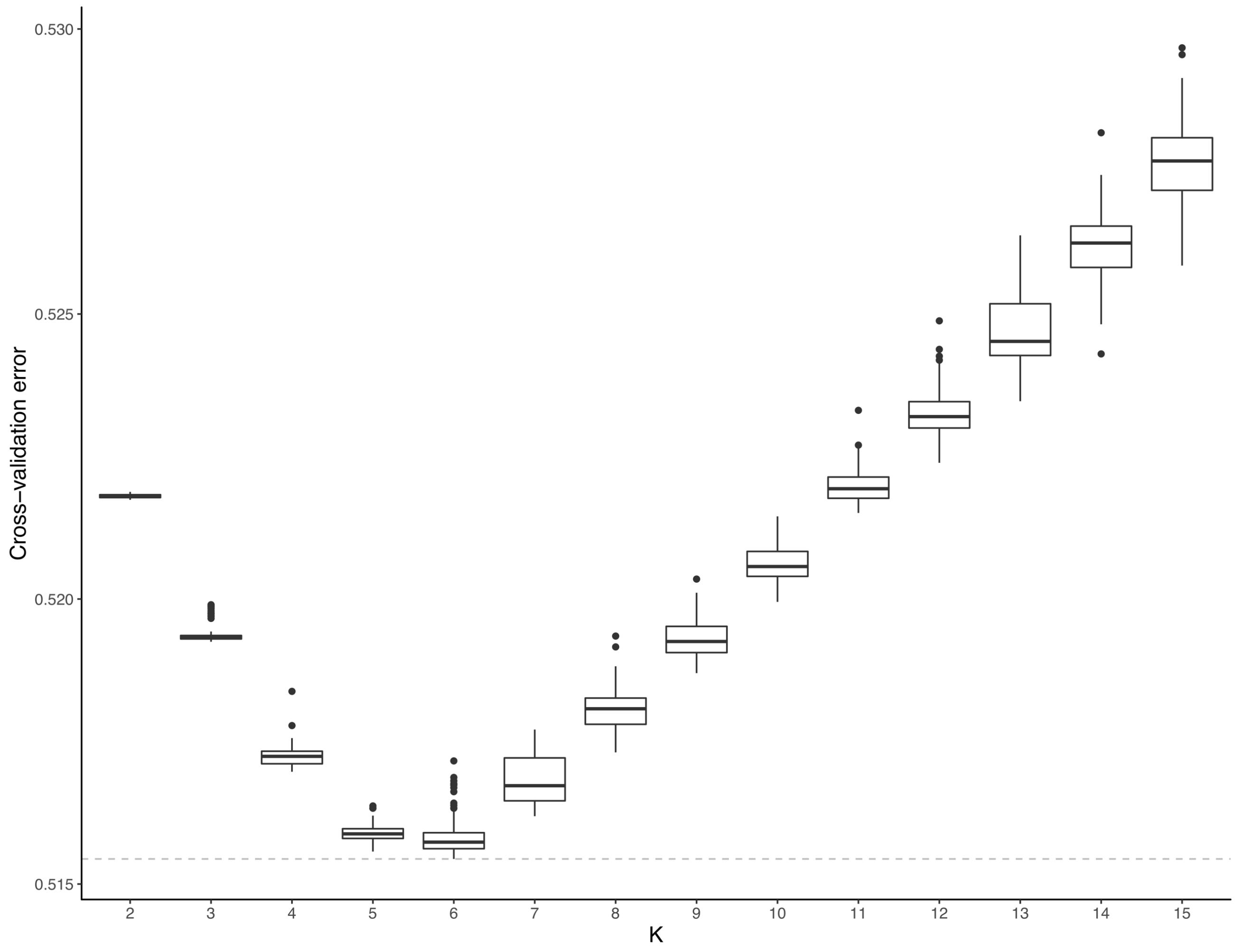

Fig. S4. Cross-validation error of ADMIXTURE runs for K= 2 to K = 15, based on 100 runs for each K value.

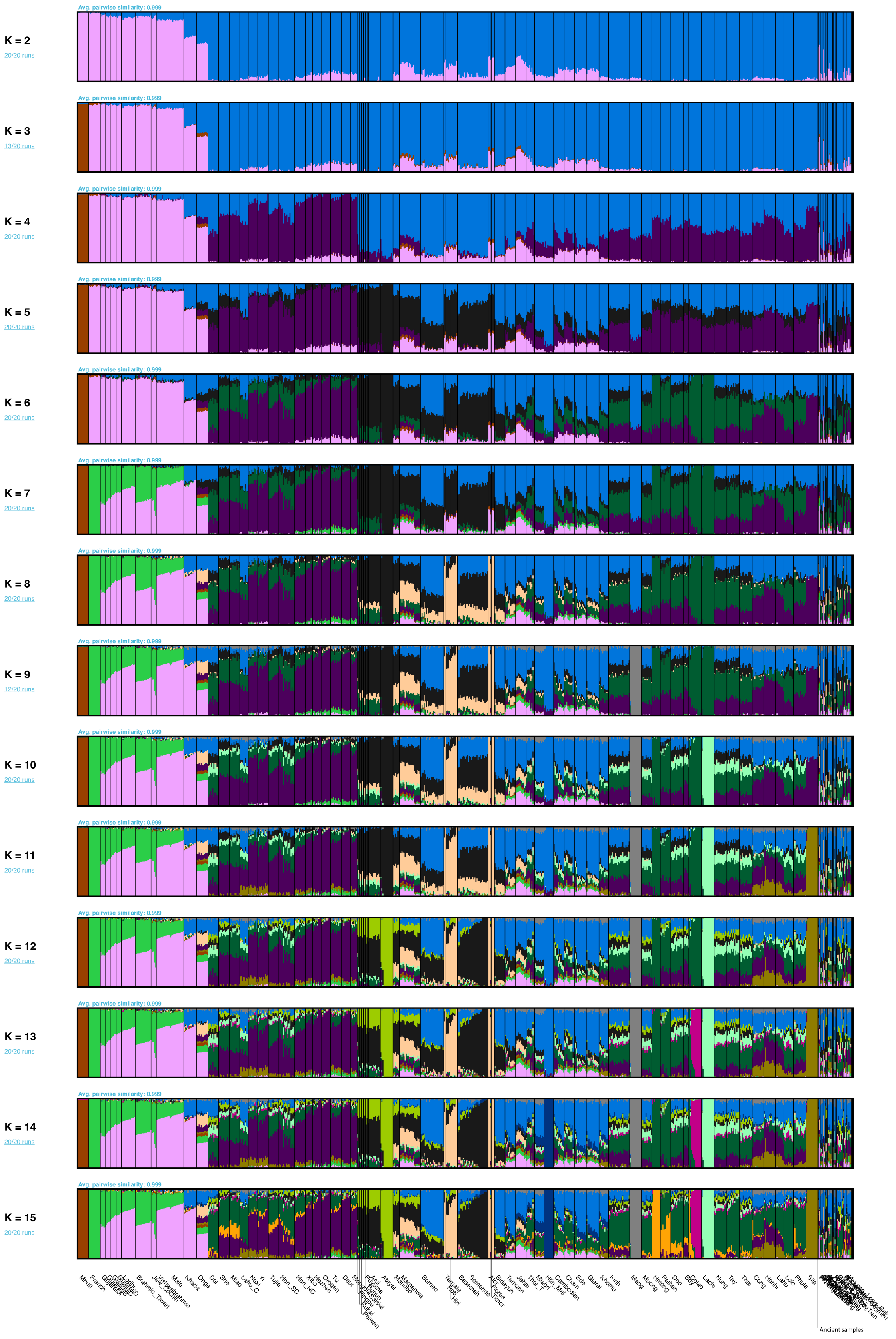

Fig. S5. ADMIXTURE results for K = 2 to K = 15.

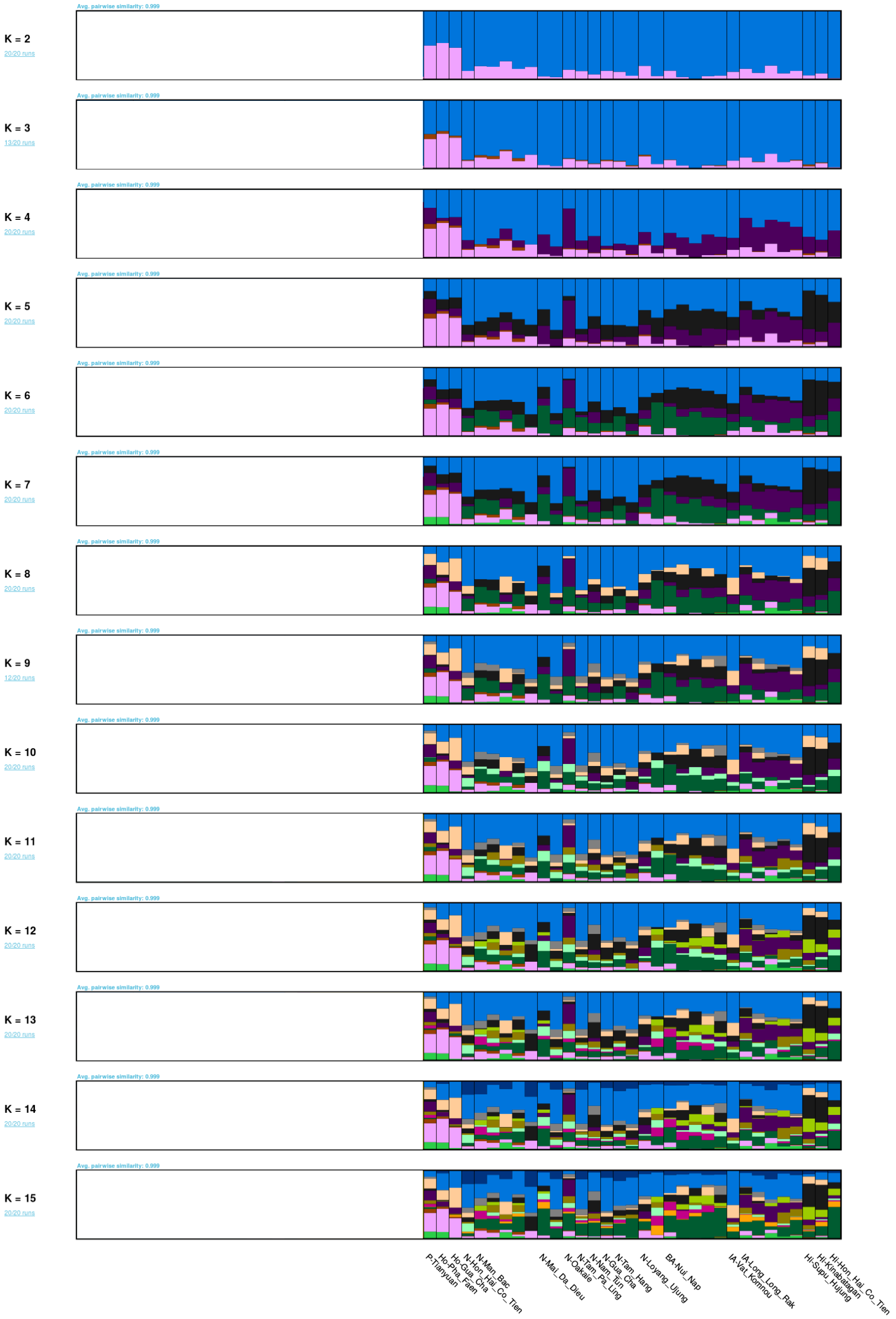

Fig. S6. ADMIXTURE results for the ancient samples for K = 2 to K = 15.

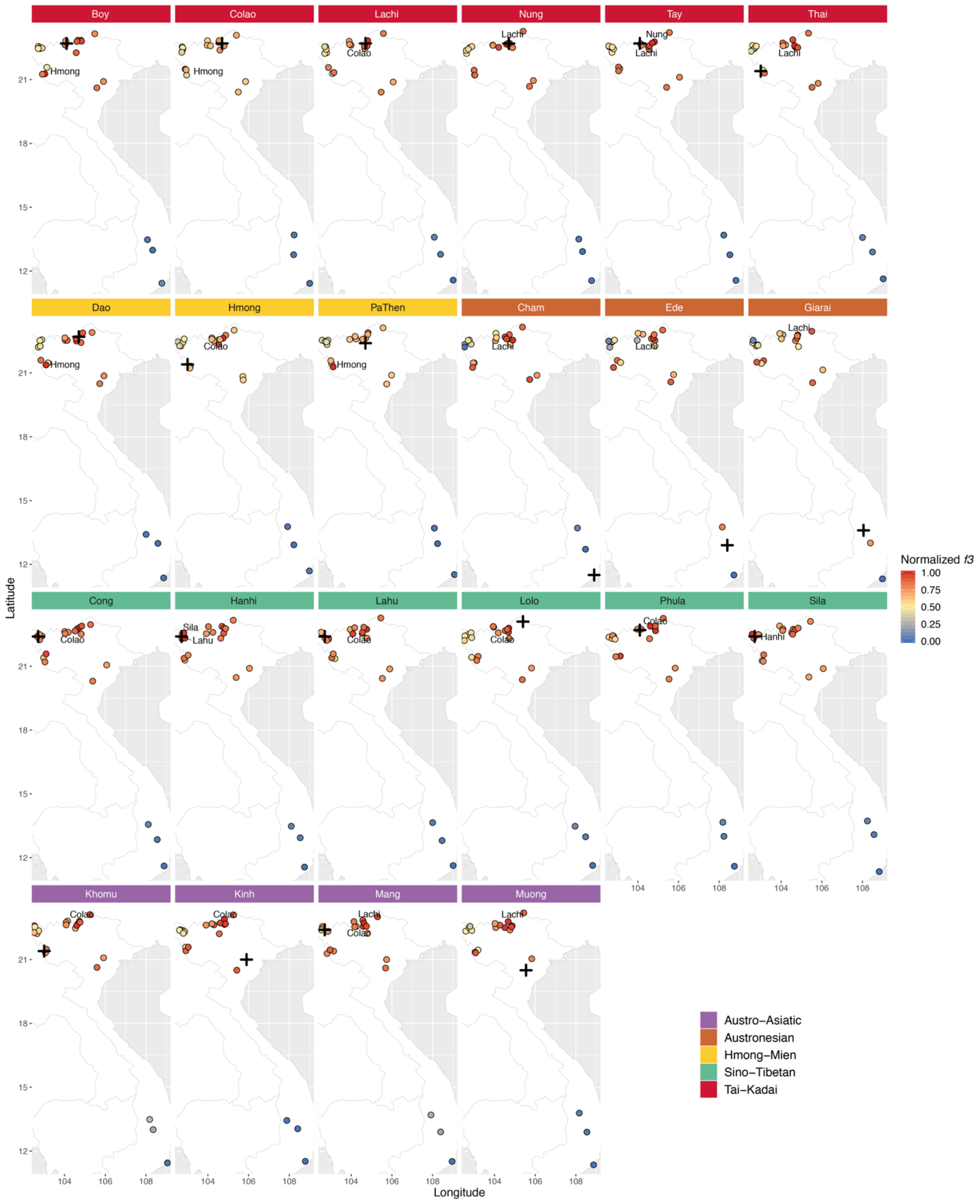

Fig. S7. Map visualization of outgroup *f3* profiles of Vietnamese ethnolinguistic groups, compared with other Vietnamese groups.

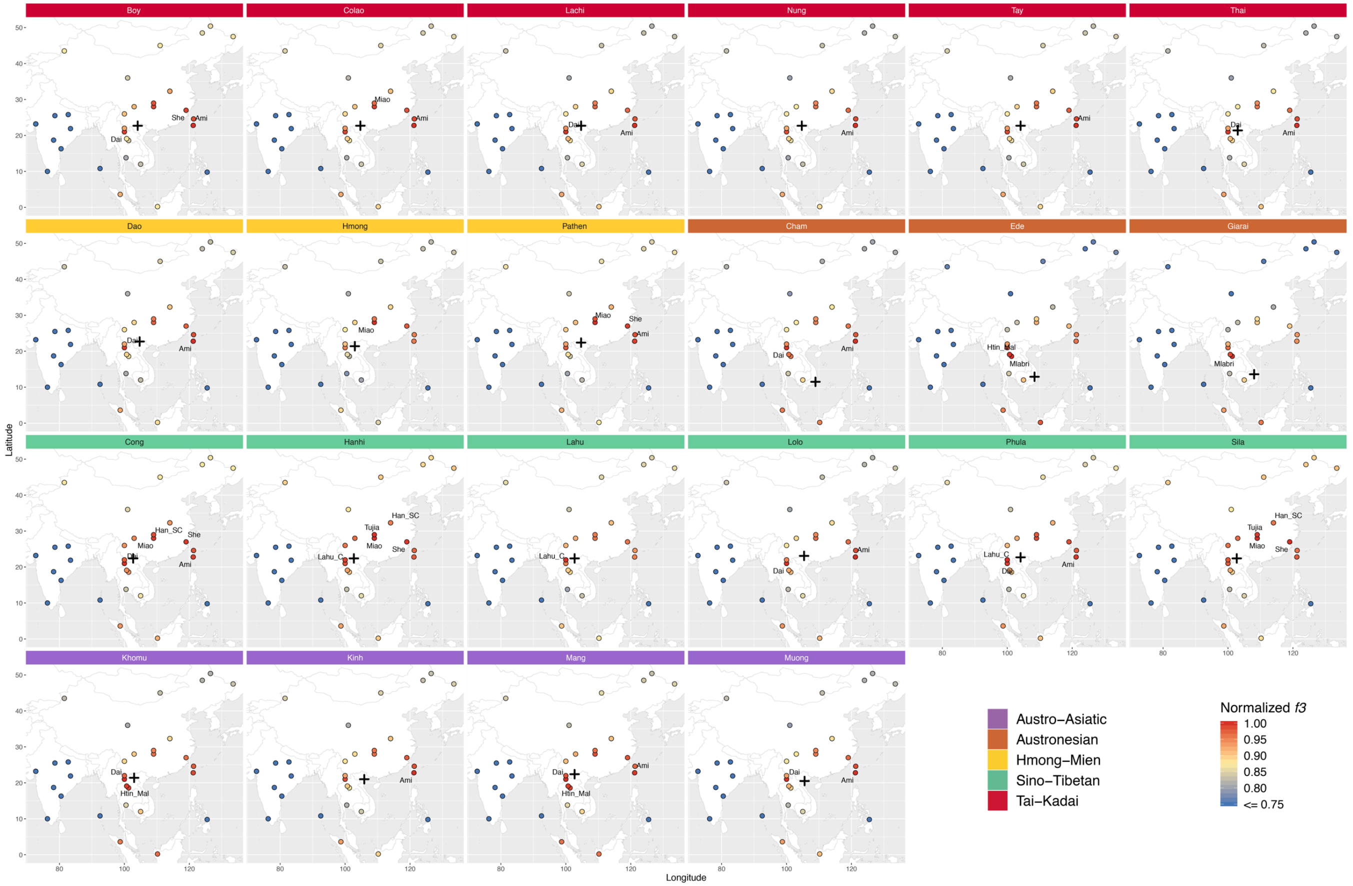

Fig. S8. Map visualization of outgroup *f3* profiles of Vietnamese ethnolinguistic groups compared with nearby modern populations (Mbuti as outgroup).

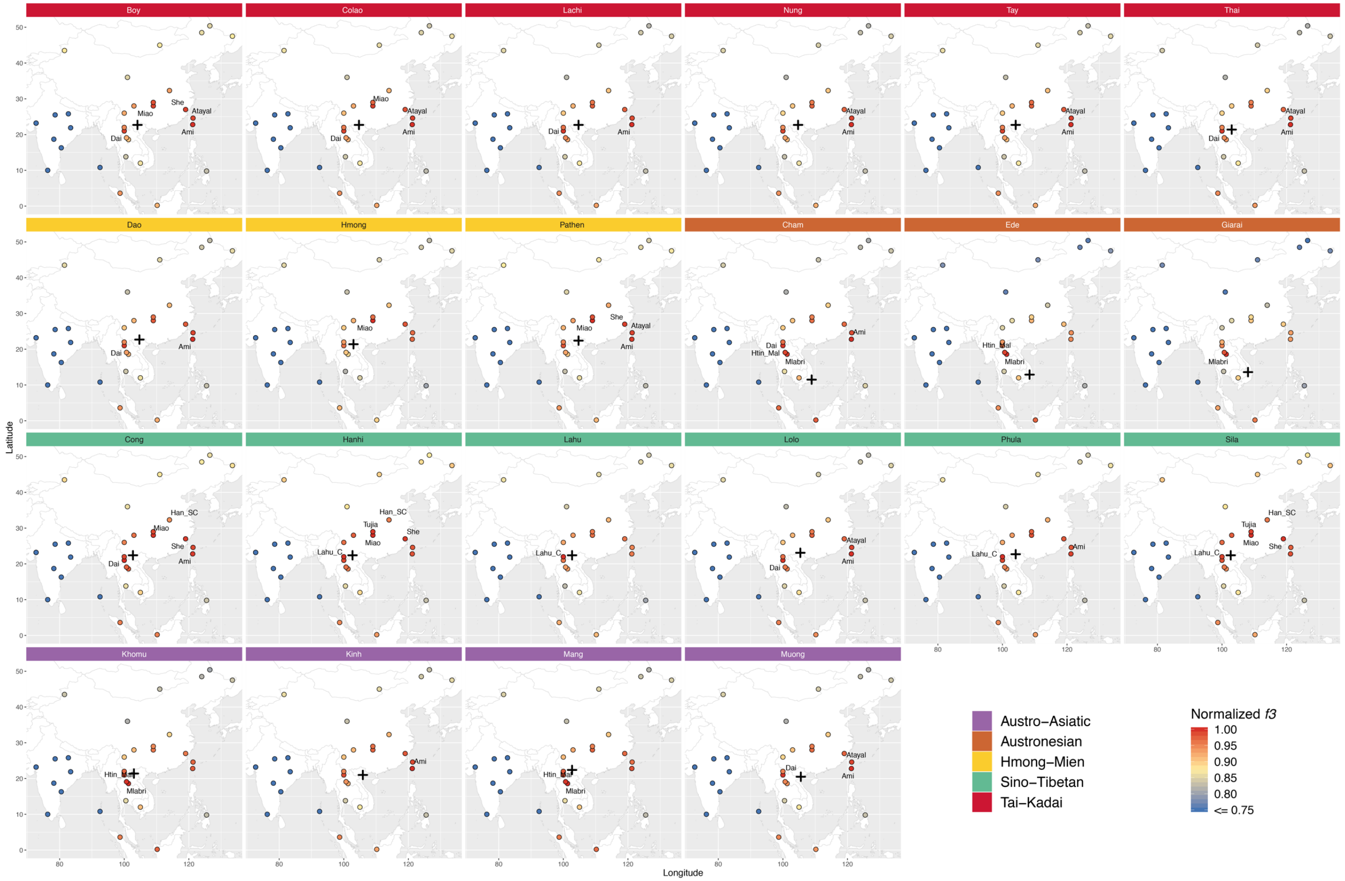

Fig. S9. Map visualization of outgroup *f3* profile of Vietnamese ethnolinguistic groups compared with nearby modern populations (French as outgroup).

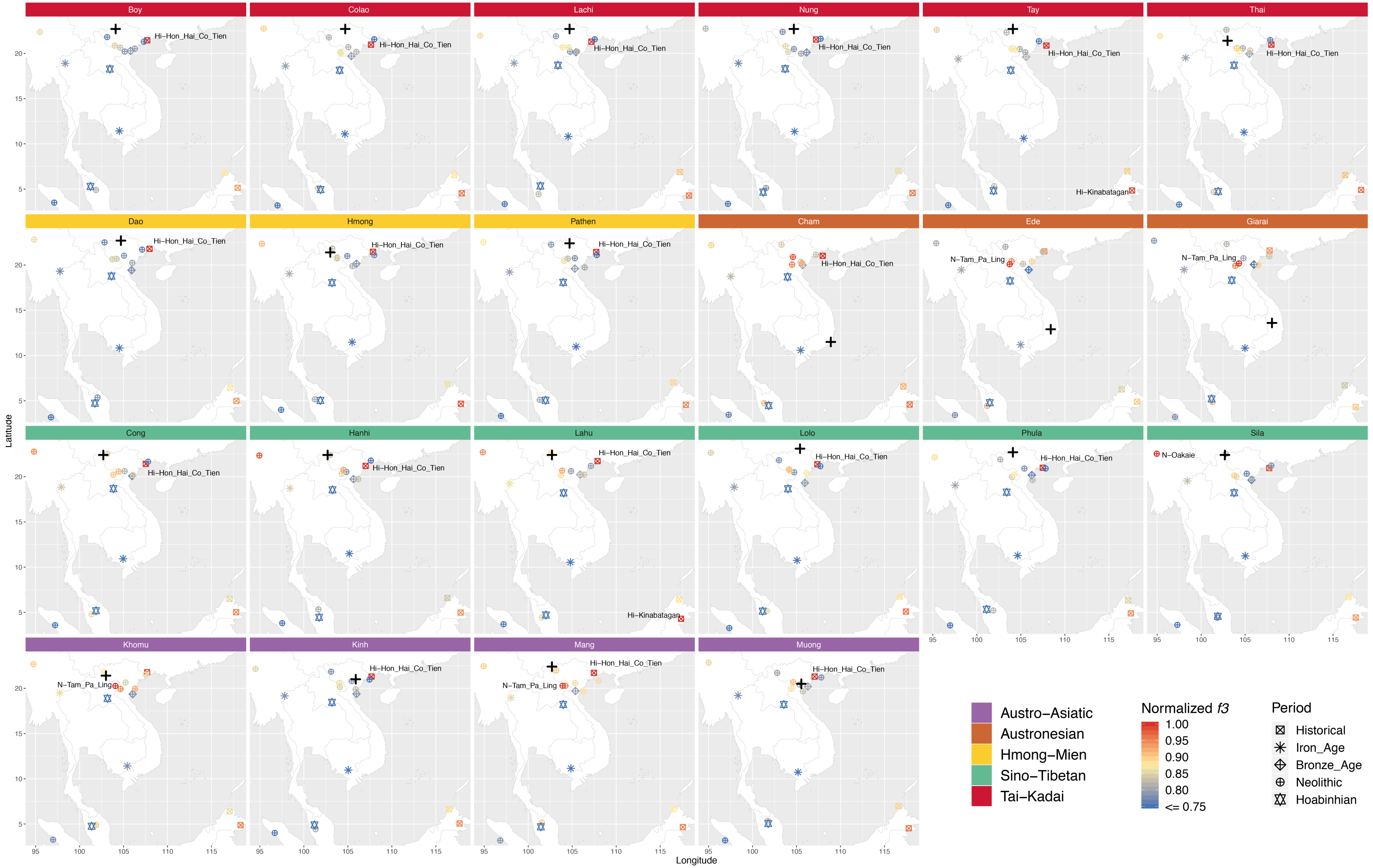

Fig. S10. Map visualization of outgroup *f3* profile of Vietnamese ethnolinguistic groups compared with the ancient samples.

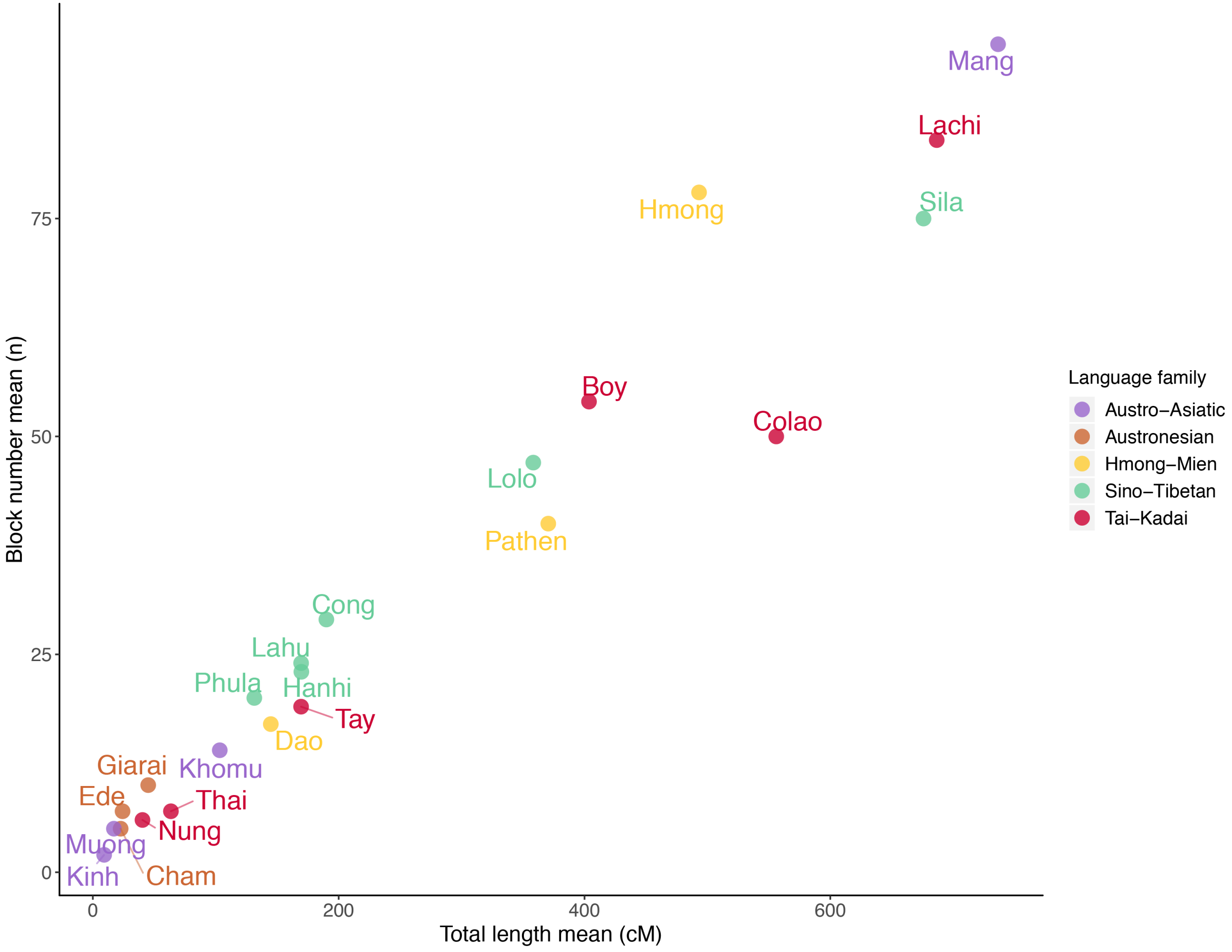

Fig. S11. IBD sharing within each Vietnamese ethnolinguistic group.

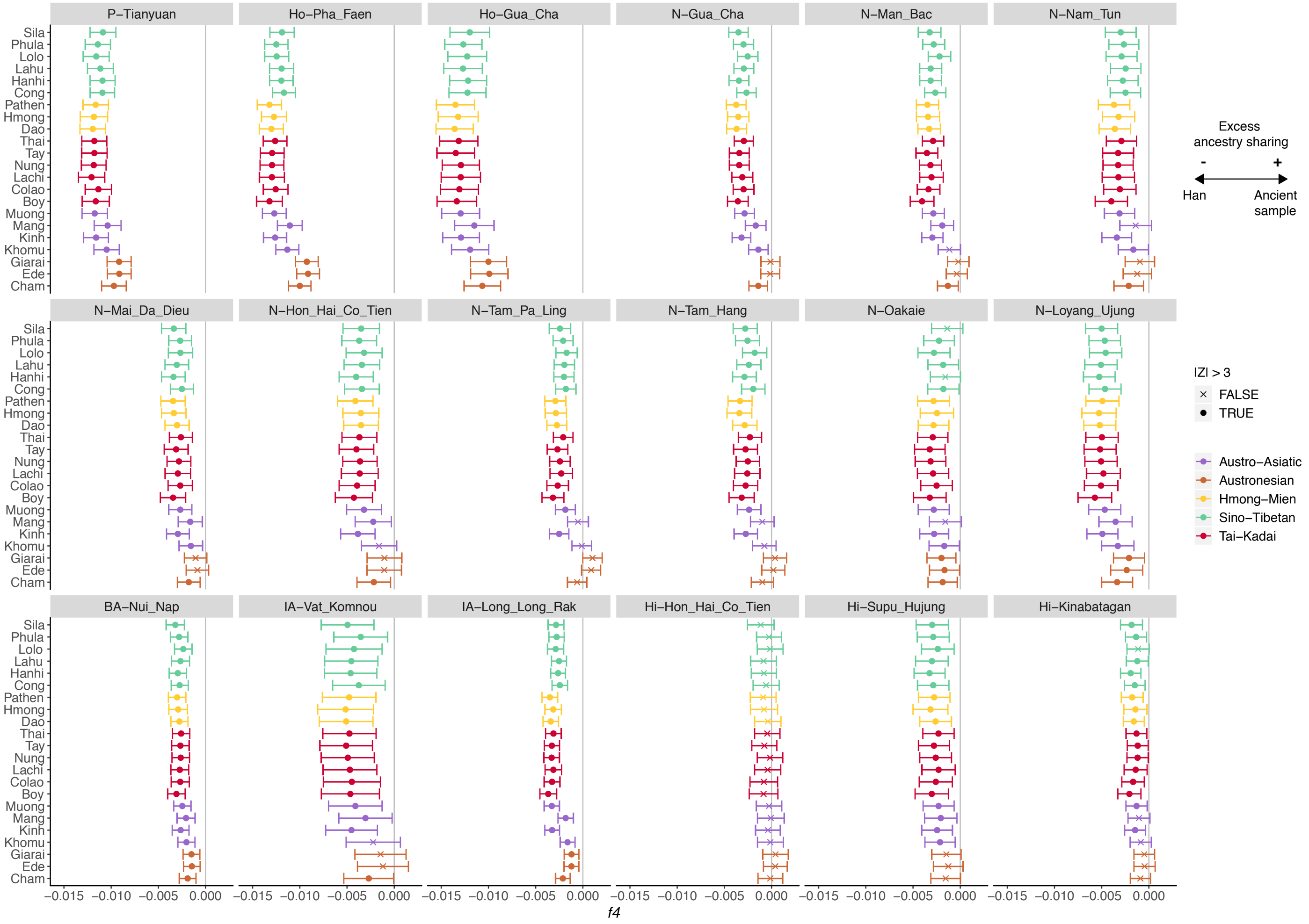

Fig. S12. ***f4*** **statistics comparing Vietnamese groups to the ancient samples.**

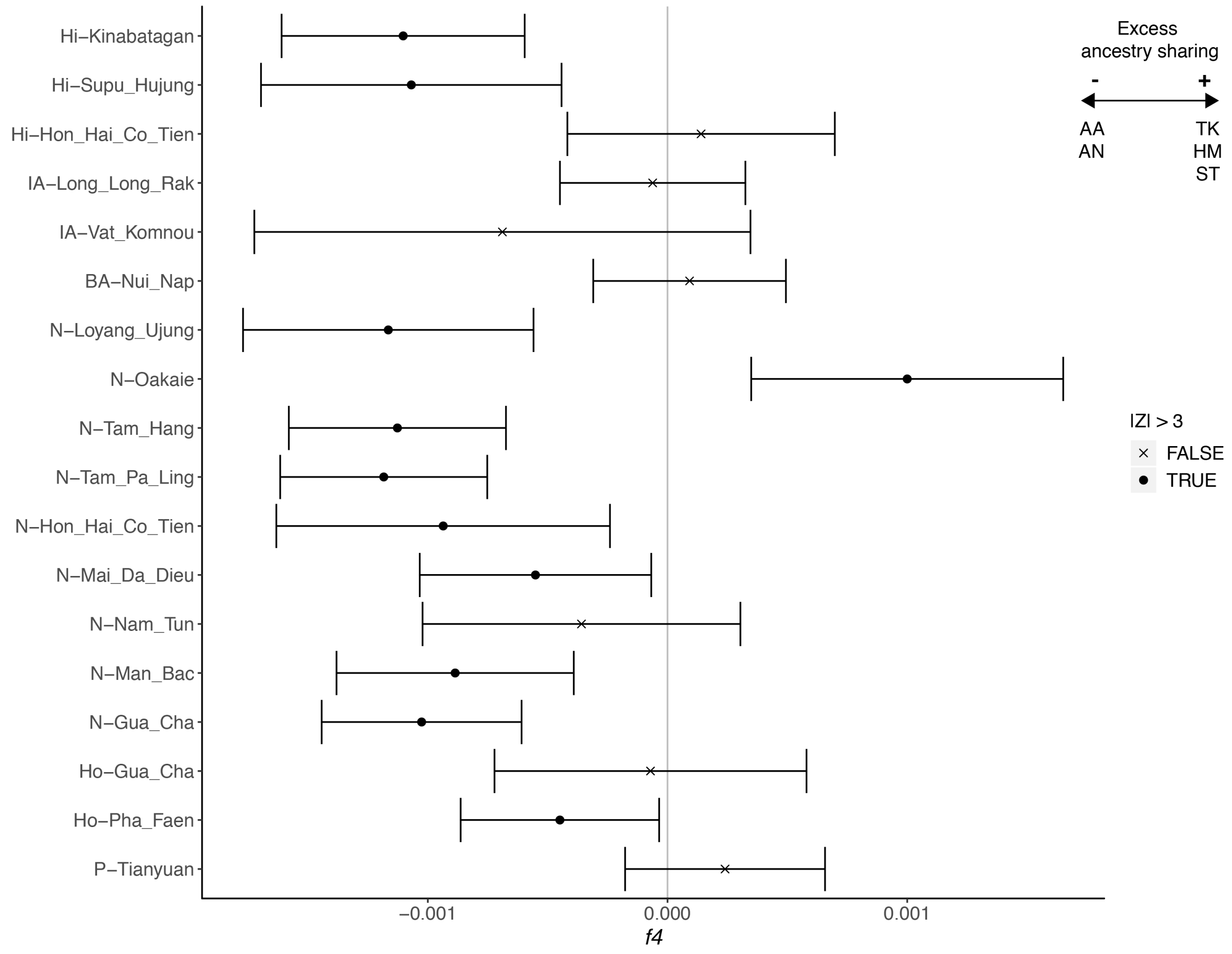

Fig. S13. ***f4*** **statistics comparing the ancient samples to the TK, HM, and ST groups and AA and AN groups.**

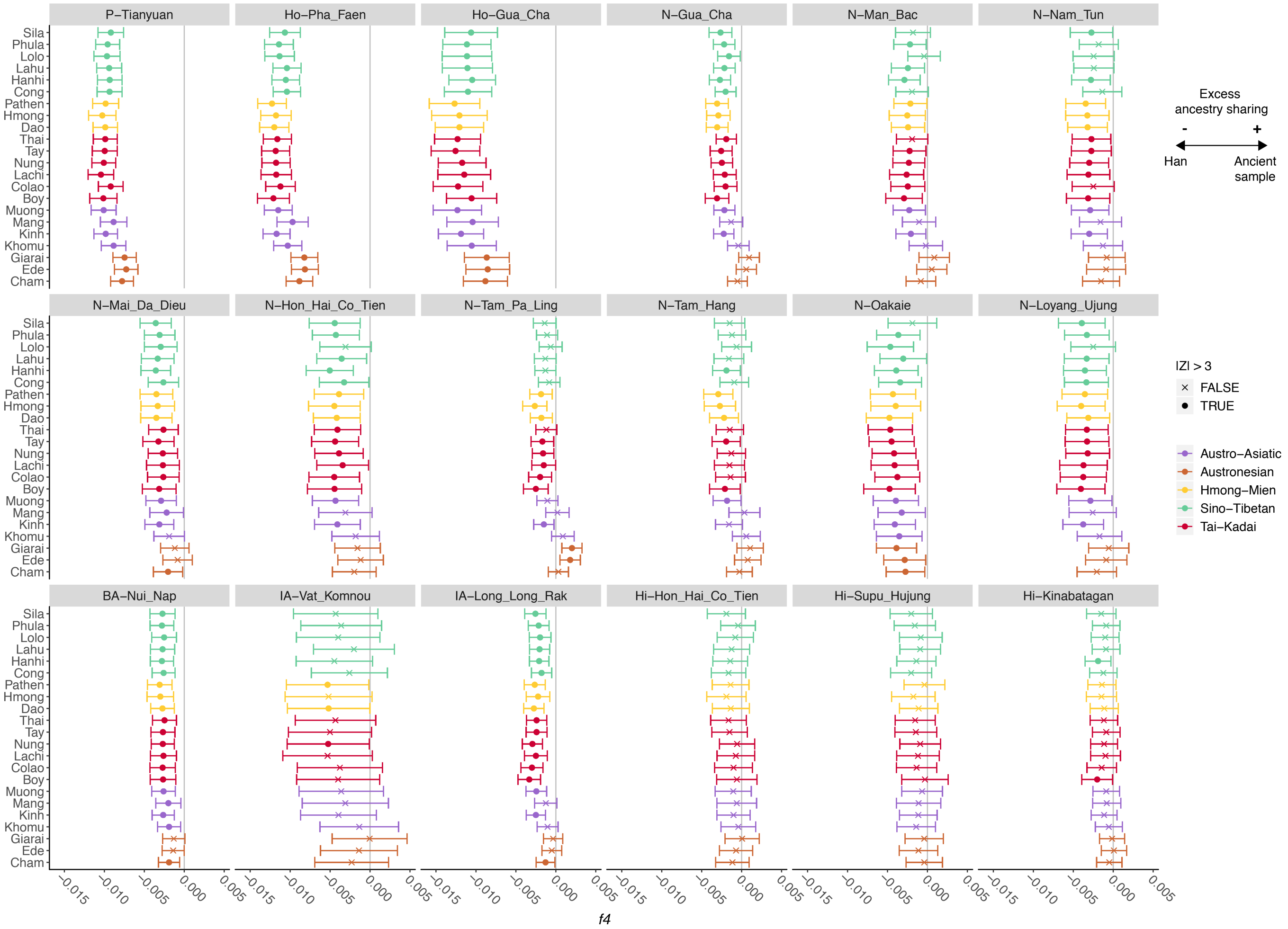

Fig. S14. ***f4*** **statistics comparing Vietnamese groups to the ancient samples, using only transversions and French as outgroup.**

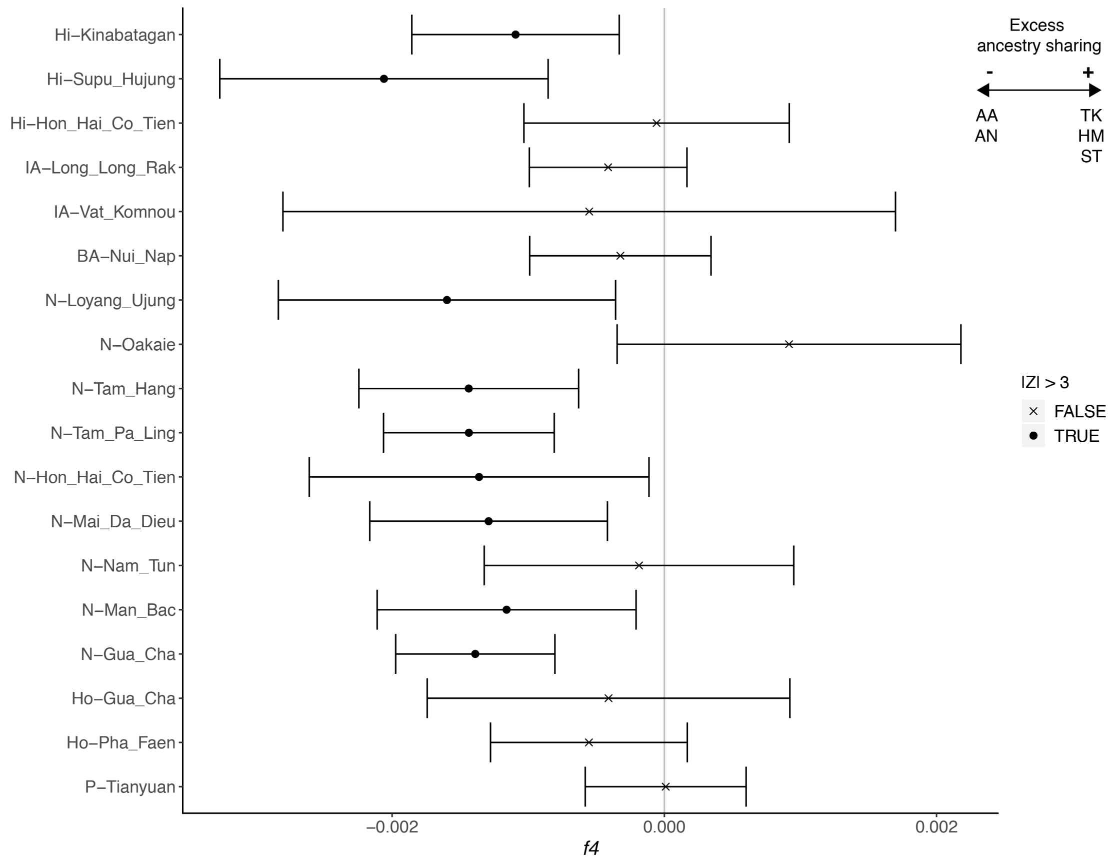

Fig. S15. ***f4*** **statistics comparing the ancient samples to the TK, HM, and ST groups and AA and AN groups, using only transversions and French as outgroup.**

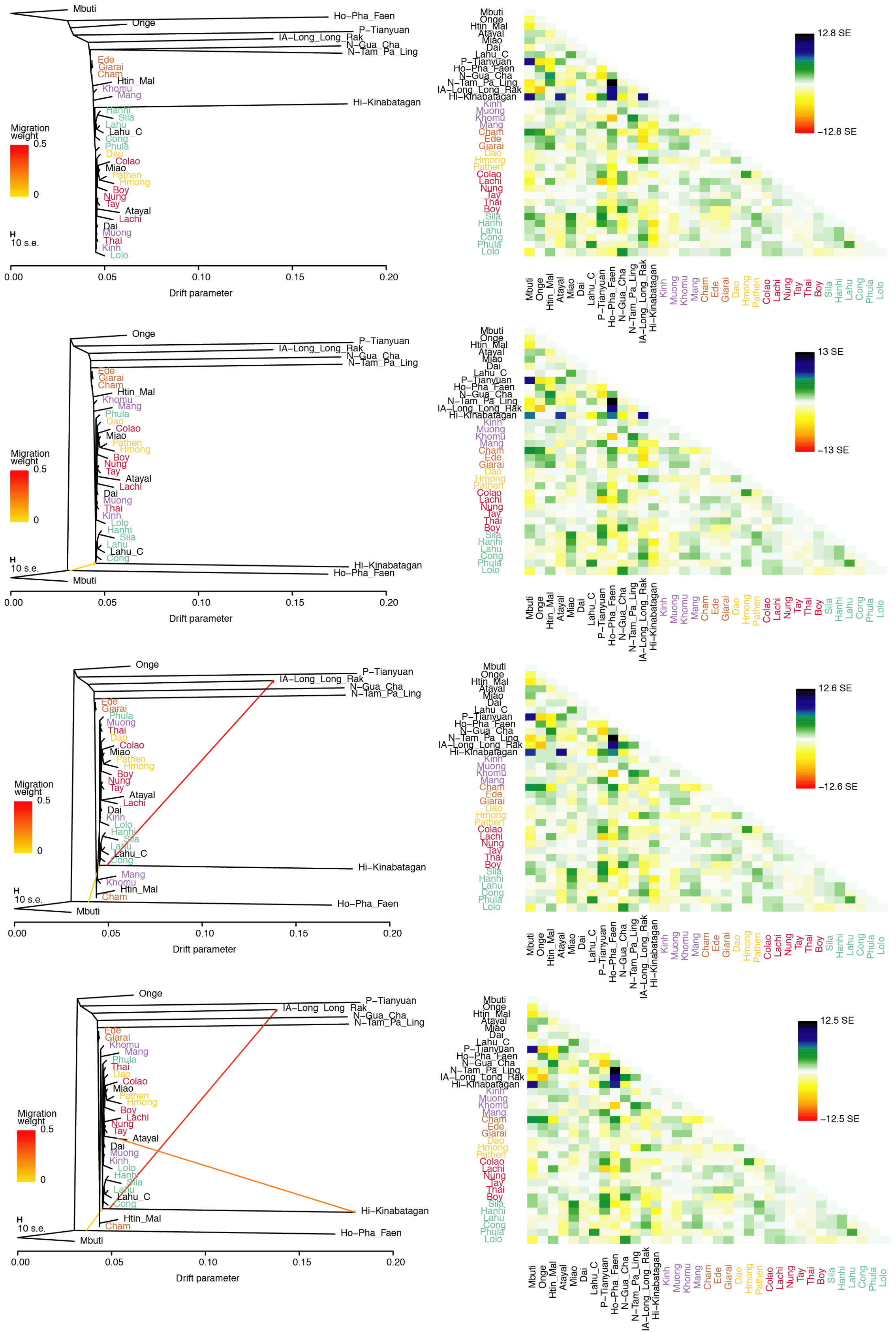

Fig. S16. Global TreeMix results with 0 to 3 migrations.

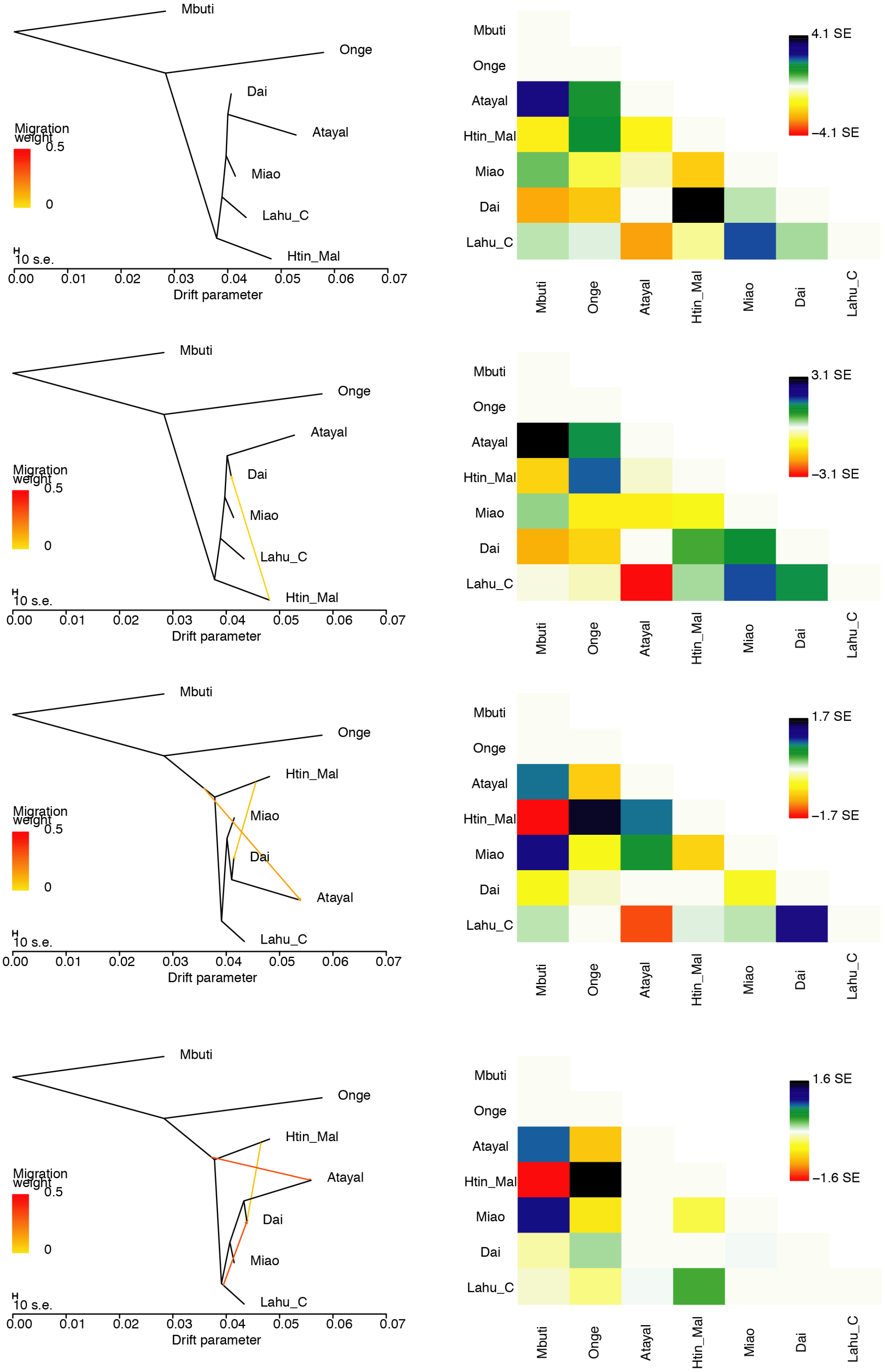

Fig. S17. TreeMix results for the backbone populations with 0 to 3 migrations.

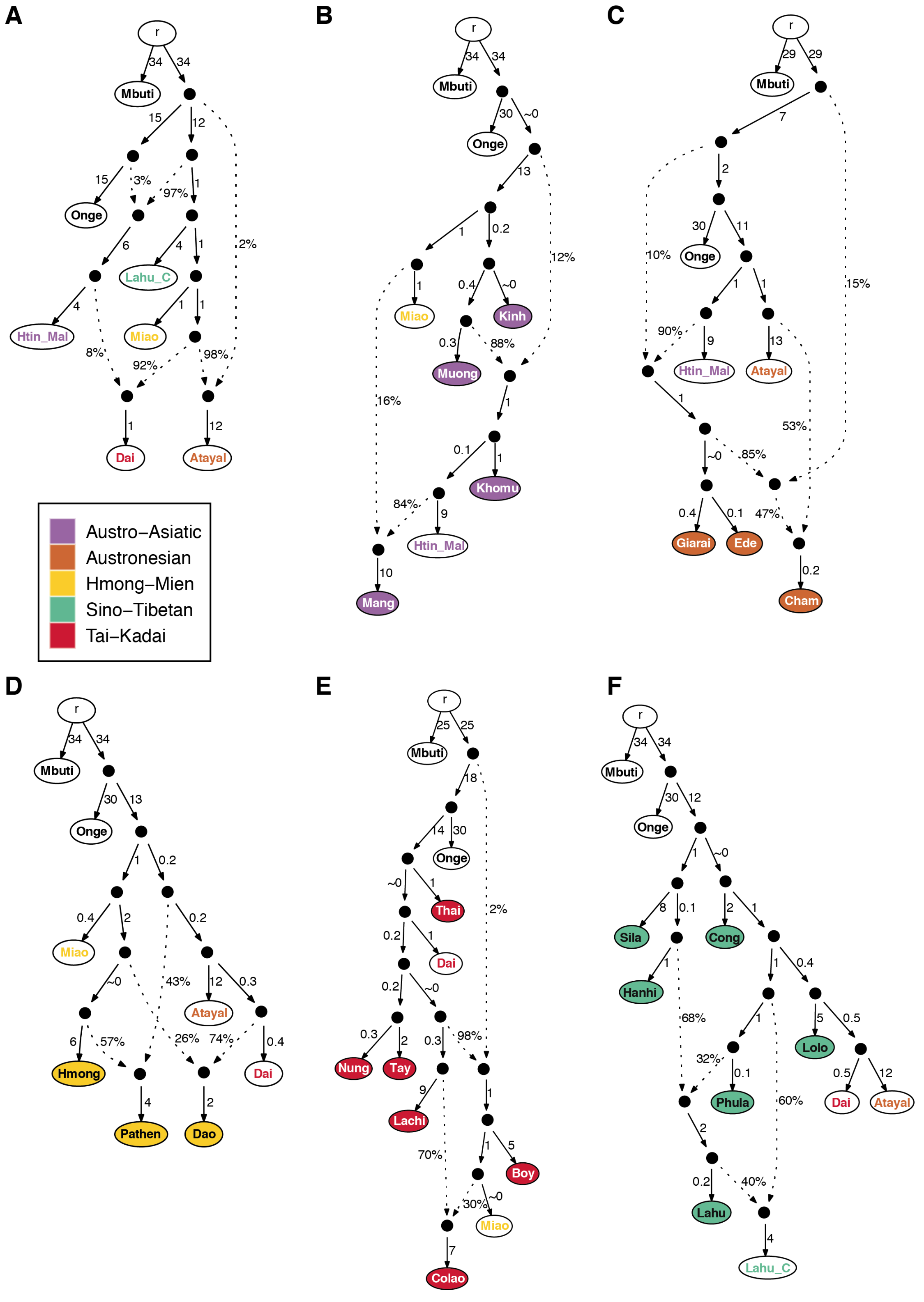

Fig. S18. Alternative admixture graphs for the Vietnamese groups, for each language family.

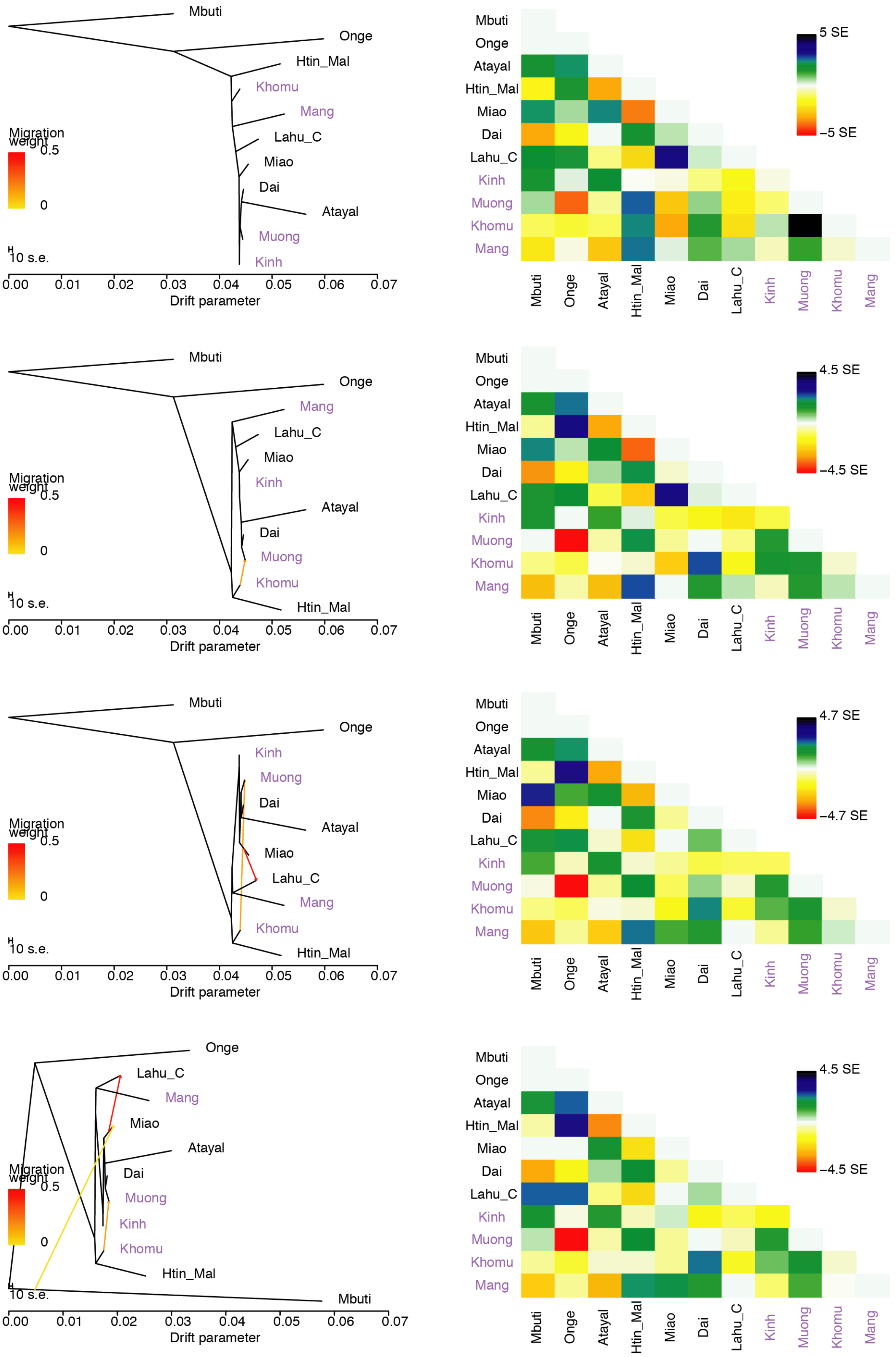

Fig. S19. TreeMix results for the Vietnamese AA groups with 0 to 3 migrations.

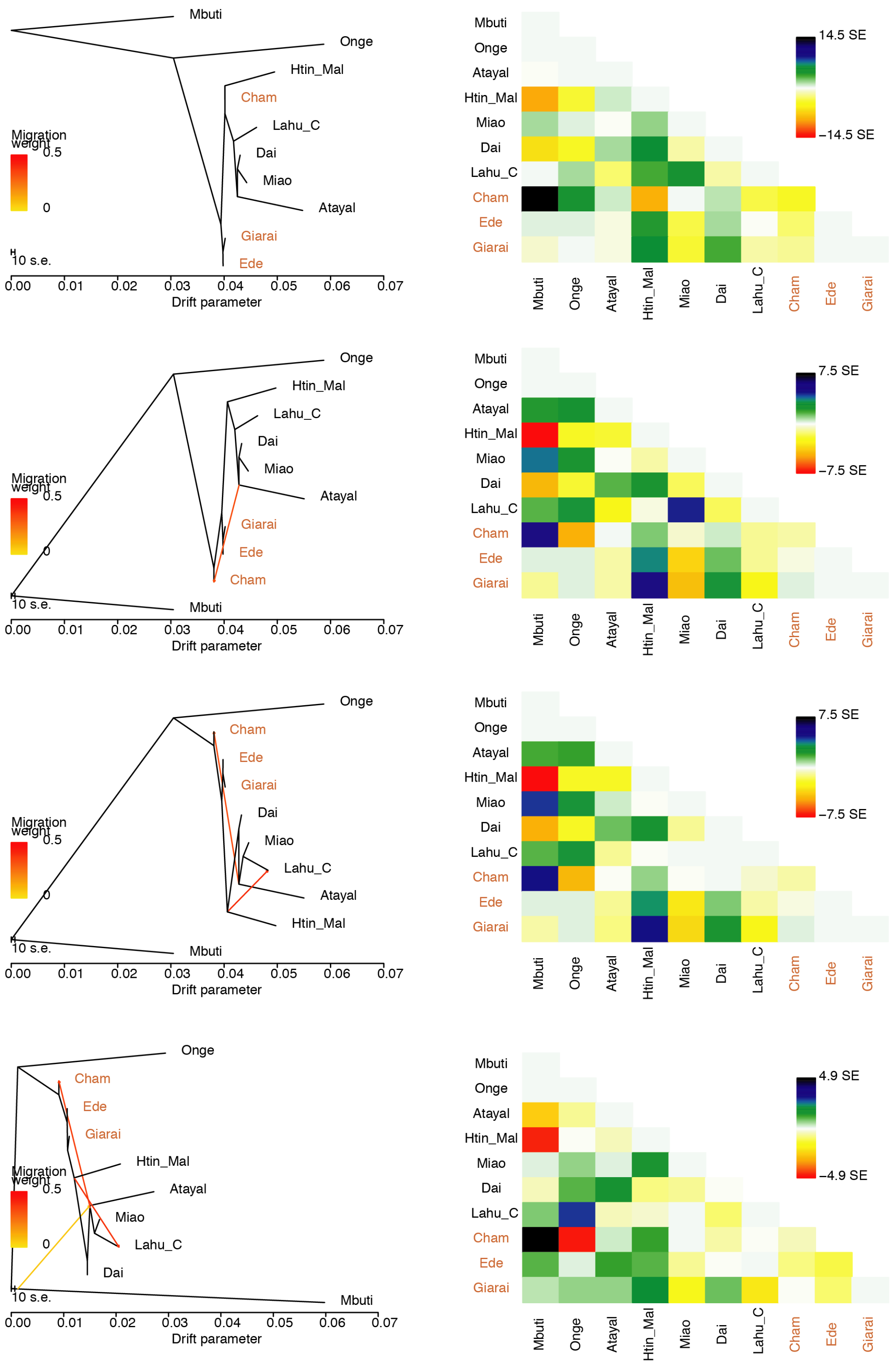

Fig. S20. TreeMix results for the Vietnamese AN groups with 0 to 3 migrations.

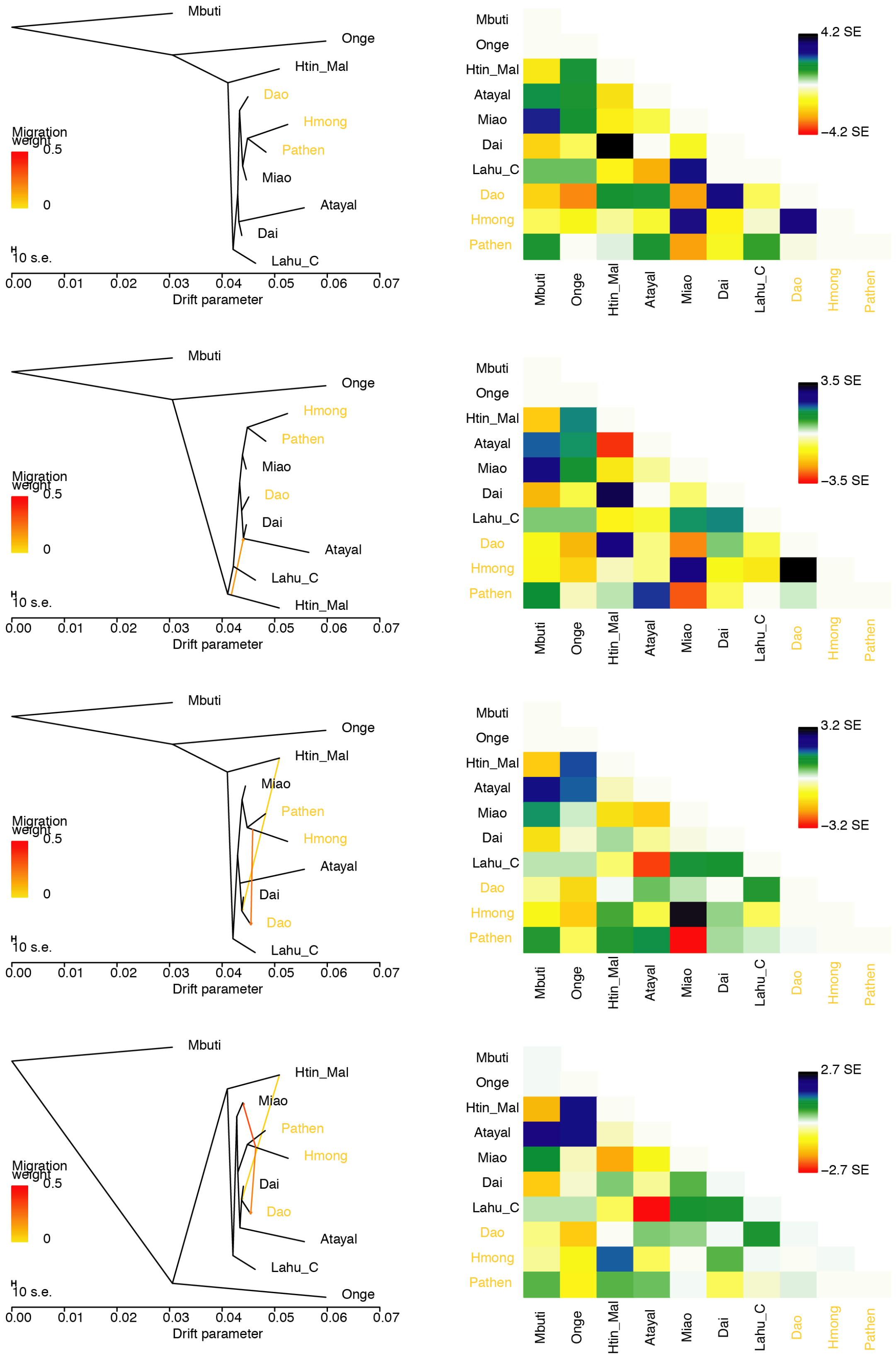

Fig. S21. TreeMix results for the Vietnamese HM groups with 0 to 3 migrations.

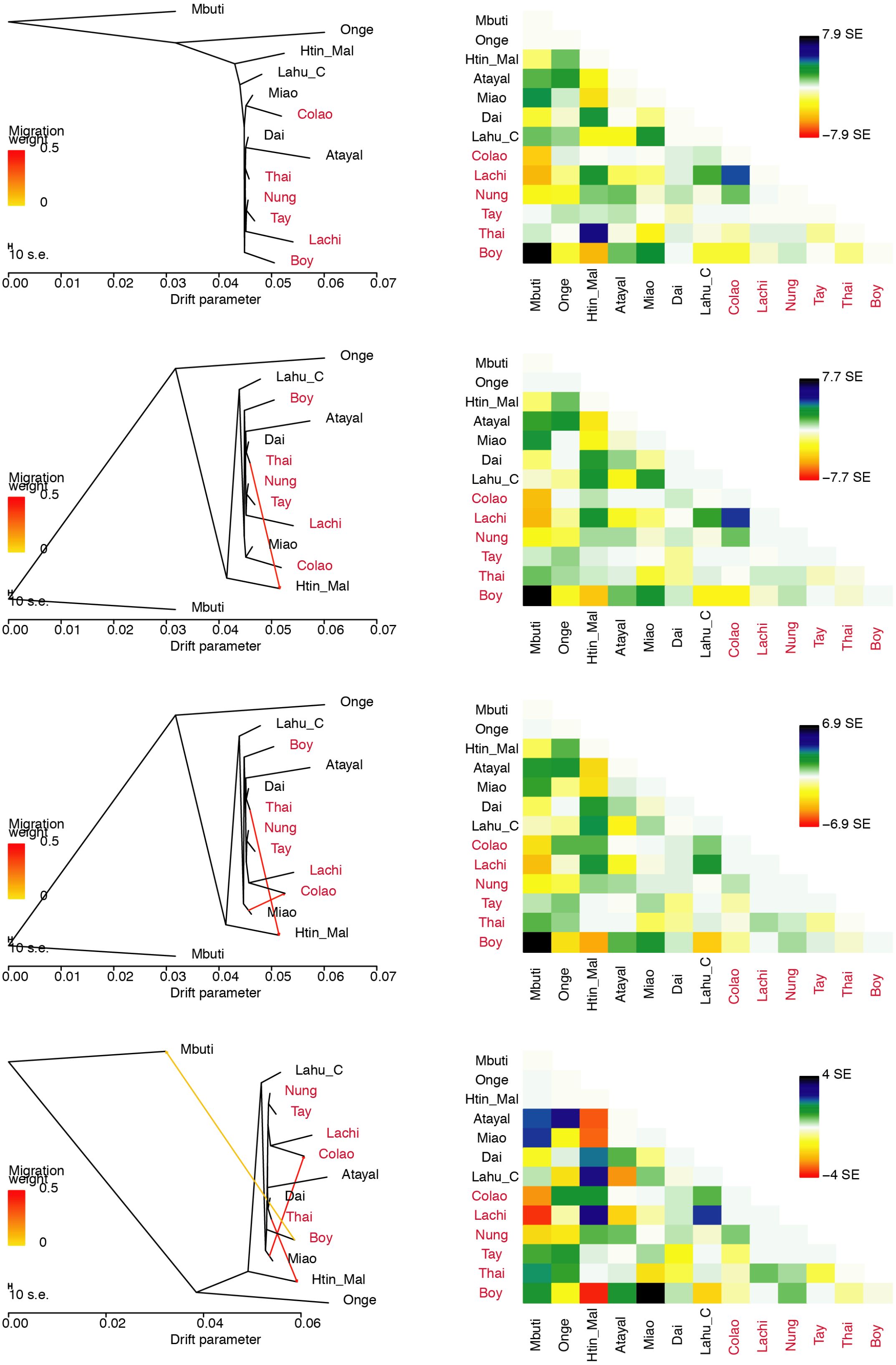

Fig. S22. TreeMix results for the Vietnamese TK groups with 0 to 3 migrations.

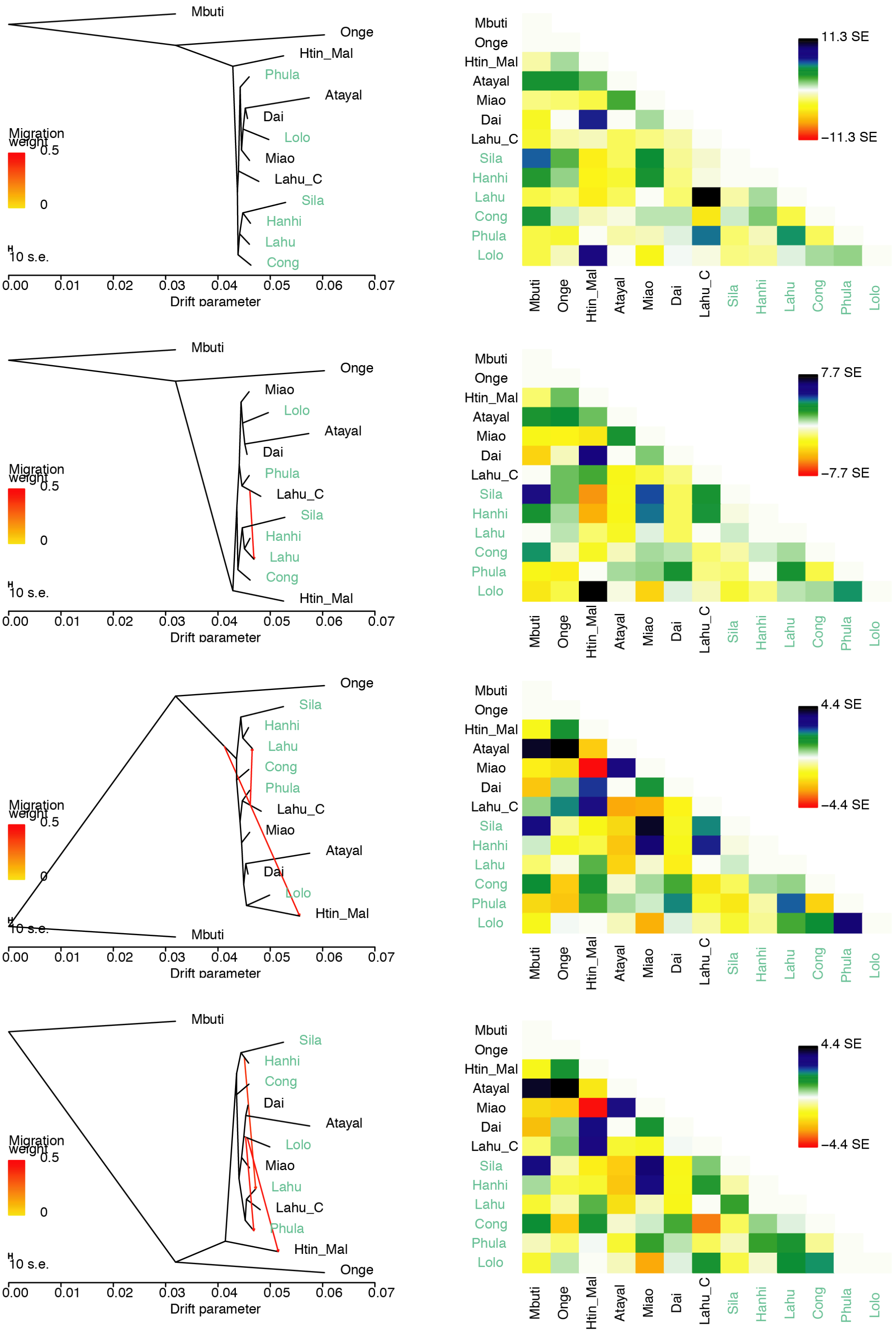

Fig. S23. TreeMix results for the Vietnamese ST groups with 0 to 3 migrations.
